# Two Novel PET Radiopharmaceuticals for Endothelial Vascular Cell Adhesion Molecule-1 (VCAM-1) Targeting

**DOI:** 10.3390/pharmaceutics13071025

**Published:** 2021-07-06

**Authors:** Sara Pastorino, Sara Baldassari, Giorgia Ailuno, Guendalina Zuccari, Giuliana Drava, Andrea Petretto, Vanessa Cossu, Cecilia Marini, Silvana Alfei, Tullio Florio, Gianmario Sambuceti, Gabriele Caviglioli

**Affiliations:** 1 Nuclear Medicine Unit, S. Andrea Hospital, via Vittorio Veneto 197, 19124 La Spezia, Italy; sara.pastorino@asl5.liguria.it; 2 Department of Pharmacy, University of Genova, viale Cembrano 4, 16148 Genova, Italy; baldassari@difar.unige.it (S.B.); ailuno@difar.unige.it (G.A.); zuccari@difar.unige.it (G.Z.); drava@difar.unige.it (G.D.); alfei@difar.unige.it (S.A.); 3 Core Facilities-Clinical Proteomics and Metabolomics, IRCCS Istituto Giannina Gaslini, via Gerolamo Gaslini 5, 16147 Genova, Italy; andreapetretto@gaslini.org; 4 Department of Health Science, University of Genova—Nuclear Medicine Unit, via A. Pastore 1, 16132 Genova, Italy; vane6291@gmail.com (V.C.); cecilia.marini@unige.it (C.M.); sambuceti@unige.it (G.S.); 5 IRCCS Ospedale Policlinico San Martino, Largo R. Benzi 10, 16132 Genova, Italy; tullio.florio@unige.it; 6 CNR Institute of Bioimages and Molecular Physiology, via Fratelli Cervi 93, 20090 Segrate, Italy; 7 Department of Internal Medicine, University of Genova, viale Benedetto XV 2, 16136 Genova, Italy

**Keywords:** radiopharmaceuticals, VCAM-1, pretargeting, imaging, PET, Gallium-68, avidin–biotin complex, inflammation, peptide

## Abstract

Atherosclerosis is a chronic progressive disease involving inflammatory events, such as the overexpression of adhesion molecules including the endothelial Vascular Cell Adhesion Molecule-1 (VCAM-1). VCAM-1 is rapidly overexpressed in the first stages of atherosclerosis, thus representing a promising target for early atheroma detection. Two novel Positron Emission Tomography (PET) radiopharmaceuticals (MacroP and NAMP), based on the VCAM-1-binding peptide having sequence VHPKQHRGGSKGC, were synthesized and characterized. MacroP is derived from the direct conjugation of a DOTA derivative with the peptide, while NAMP is a biotin derivative conceived to be employed in a three-step pretargeting system, involving the use of a double-chelating derivative of DOTA. The identity of the newly synthesized radiopharmaceuticals was confirmed by mass spectrometry and, after radiolabeling with ^68^Ga, both showed high radiochemical purity; in vitro tests on human umbilical vein endothelial cells evidenced their VCAM-1 binding ability, with higher radioactive uptake in the case of NAMP. Moreover, NAMP might also be employed in a theranostic approach in association with functionalized biotinylated nanoparticles.

## 1. Introduction

Atherosclerosis is the most diffused cardiovascular disease in developed countries [1]. It is a chronic progressive disease characterized by the subendothelial accumulation of lipids, immune cells, foam cells (i.e., cholesterol-engorged macrophages), fibrous elements, platelets and extracellular matrix, forming the atherosclerotic plaque [2,3]. Atherosclerotic plaques reduce the vessel lumen and, in the case of vulnerable plaques (i.e., plaques prone to rupture), can cause thrombosis. A prominent role in atherosclerosis and its complications is attributed to inflammation [4,5]. In particular, the relevance of the overexpression of endothelial adhesion proteins in the early stages of atherosclerosis development was firstly demonstrated by the observation that cholesterol accumulation in the vasal intima induced the increased expression of VCAM-1 on endotheliocyte membranes [6].

VCAM-1 is a transmembrane protein, expressed by endotheliocytes and other cell types under the stimulation of cytokines such as TNF-α, IL-1β and IL-4 [7]. This protein is involved in the interaction between inflamed endothelium and circulating leukocytes, by mediating the tethering and firm adhesion of leukocytes, preceding diapedesis. VCAM-1 has attracted a great deal of interest as a potential target for inflammation detection and, consequently, for atherosclerosis diagnosis, because it is scarcely expressed by healthy endothelium, while being rapidly upregulated in damaged and lesion-predisposed vascular regions [8]. Moreover, VCAM-1 is readily accessible to blood-borne targeted contrast agents.

A wide variety of strategies for VCAM-1 targeting have been explored, as reported in recent review papers [9,10]. Antibodies and their fragments are frequently used for targeting purposes, but they are affected by several drawbacks such as immunogenicity and capture by the mononuclear phagocyte system [11]. Another way to target VCAM-1 is to exploit peptides that are characterized by high affinity and specificity for VCAM-1 binding: several research groups developed VCAM-1 targeting systems for the diagnosis and therapy of atherosclerosis and cancer, by exploiting VCAM-1 targeting peptides [10]. Among the VCAM-1-binding peptides already described in the literature, the sequence VHPKQHR discovered by in vivo phage display by Kelly et al. [12] is very promising due to its high affinity. In most work employing this peptide, the VCAM-1 binding sequence was elongated with different linkers [9], such as the GGSKGC sequence [12]; indeed, the linker allows for the conjugation of the VCAM-1 binding sequence without hampering its affinity for the target.

Pretargeting is a strategy consisting of the decoupling of a targeting agent, usually a modified monoclonal antibody, and a small radiolabeled molecule [13]. In vivo pretargeting includes different steps: the first is the injection of the targeting agent, followed by a stage during which the targeting molecules accumulate at the target and the unbound ones are cleared from blood circulation; then, a radioligand binding to the targeting agent is administered. The radioligand, due to its small dimensions, is usually characterized by favorable pharmacokinetics, ensuring its rapid distribution and excretion and consequently reducing the background signal [14]. The procedure might also include one or two chasing steps: for example, after the first injection, an appropriate anti-targeting agent can be administered in order to accelerate the elimination of unbound targeting ligand from the blood. This kind of systems present several advantages, such as decreased circulation time of radioactivity within the body, reduced uptake of radioactives by healthy tissues, shorter time to achieve adequate tumor-to-background ratios, with the possibility of employing short-lived radionuclides, as ^68^Ga [13,14]. In pretargeting systems, the strong interaction and high affinity (Kd = 10^−15^ M) of the biotin/avidin system is frequently exploited [15].

**Figure 1:**
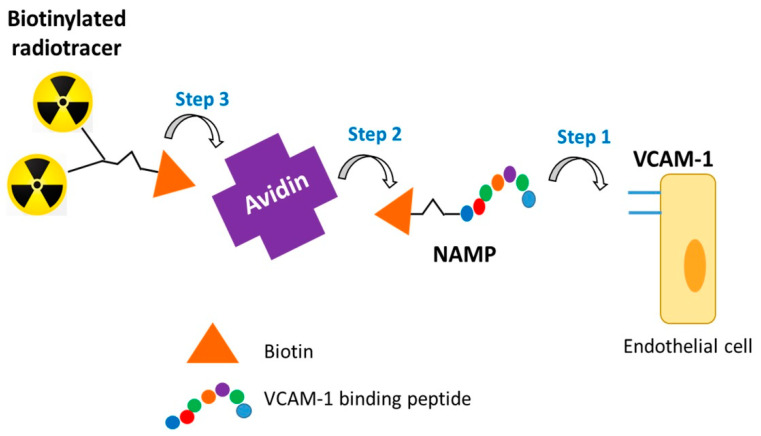
Three-step pretargeting scheme.

In this work, the synthesis and characterization of two novel PET radiotracers, based on the VHPKQHRGGSKGC peptide, are described. The first one, named MacroP, is obtained from the reaction of a DOTA (1,4,7,10-tetraazacyclododecane-1,4,7,10-tetraacetic acid) derivative with the thiol group of the cysteine of the VCAM-1-binding peptide. The second one, named NAMP, is a biotin derivative conceived to be used in a three-step pretargeting system based on the biotin/avidin high affinity complex (Figure 1). The newly synthesized molecules, after labeling with ^68^Ga, were tested on human endothelial cells overexpressing VCAM-1.

## 2. Materials and Methods

### 2.1. Synthesis

#### 2.1.1. General Considerations

All chemicals were purchased from commercially available suppliers and were used without further purification. The water used was purified with a Milli-Q system (Merck Life Science S.r.l., Milan, Italy). Semi-preparative reverse phase HPLC (RP-HPLC) was performed on a Waters 1525 binary pump HPLC with a Waters 2992 photodiode array detector, equipped with Waters Empower^TM^ 3 software, using a RP18, Waters X-Bridge C18, 5 µm, 10 × 150 mm and a Waters X Bridge Prep C18, 5 µm, 10 × 10 mm Guard Cartridge. The pure product collections were performed using a Waters fraction collector III (Waters Spa, Sesto San Giovanni, Italy). FTIR spectra were acquired as KBr pellets on a PerkinElmer System 2000 spectrophotometer (PerkinElmer, Inc., Waltham, MA, USA) interfaced to a personal computer, operating under Turbochrome workstation (version 6.1.1., PerkinElmer, Inc., Waltham, MA, USA). ^1^H and ^13^C NMR spectra were acquired on a Bruker Avance DPX 300 spectrometer (Bruker Italia S.r.l., Milan, Italy) at 300 and 75.5 MHz. Fully decoupled ^13^C NMR spectra were reported. Chemical shifts were reported in ppm (parts per million) units relative to the internal standard tetramethylsilane (TMS = 0.00 ppm), and the splitting patterns were described as follows: s (singlet), d (doublet), t (triplet), q (quartet), m (multiplet), and bs (broad signal). In addition, signal attribution was made indicating the reference group and, when possible, the number of protons was reported on the chemical structure (see Supplementary Materials) as H-1, H-2, etc. Analytical RP-HPLC was performed on Hewlett Packard Series II 1090 liquid chromatograph (HP Italy S.r.l., Cernusco Sul Naviglio, Italy) with UV-visible detector, equipped with HPLC ChemStation software, using a RP18, Waters X-Bridge C18, 3.5 µm, 4.6 × 150 mm and a Waters X-Bridge BEH C18 Sentry Guard Cartridge 3.5 µm, 4.6 × 20 mm. Mass spectrometry was performed on the mass spectrometer LTQ-Orbitrap Velos Pro (Thermo Fisher Scientific, Waltham, MA, USA), operated in positive ionization mode; single MS survey scans were performed in the Orbitrap (Thermo Fisher Scientific, Waltham, MA, USA).

#### 2.1.2. Synthesis of MacroP

**Figure 2:**
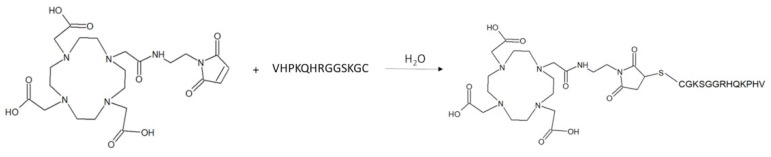
Reaction scheme of MacroP synthesis.

MacroP was synthesized as described in the reaction scheme depicted in Figure 2.

In a 1.5 mL polypropylene tube, 1.13 mg of maleimido-mono-amide-DOTA (MMA-DOTA) (1.4 μmol) were dissolved in 500 μL of Milli-Q water. VHPKQHRGGSKGC peptide (1 mg, 0.72 μmol) was solubilized in 500 μL of Milli-Q water and added to the MMA-DOTA solution. The mixture was heated at 37 °C, under orbital shaking at 400 rpm for 48 h, under nitrogen atmosphere. The product was purified by semi-preparative RP-HPLC using a gradient solvent mixture composed of 0.1% aqueous trifluoroacetic acid (TFA) (solvent A) and acetonitrile (solvent B). Linear gradient from 6% to 16% B in 10 min was applied, at 3 mL/min flow rate. The elution fraction, corresponding to MacroP, was dried under vacuum, re-dissolved in ultrapure water and analyzed by analytical RP-HPLC using the same gradient solvent mixture at 1 mL/min flow rate. The product was finally characterized by mass spectrometry using an LTQ-Orbitrap Velos Pro mass spectrometer operated in positive ionization mode, recording a mass window between 150 and 2000 *m*/*z*, with a full scan resolution set at 120,000. Samples were diluted 1:100 in water:acetonitrile (50:50 *v*/*v*) added with 1% acetic acid and introduced in the mass spectrometer at 5 μL/min flow rate with a syringe pump. MacroP chelating efficiency was determined by colorimetric method [16] described in the Supplementary Materials.

#### 2.1.3. Synthesis of NAMP

**Figure 3:**
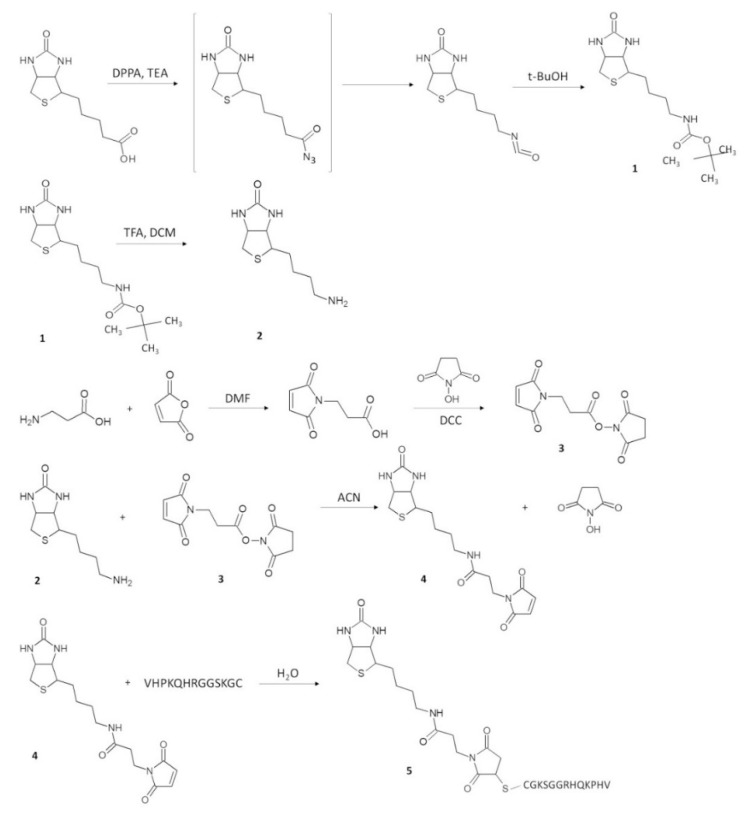
Reaction scheme of NAMP synthesis.

NAMP was synthesized by following the reaction scheme depicted in Figure 3.

N-(tert-butoxycarbonyl)-norbiotinamine (NBA-BOC) (**1**). Biotin (500 mg, 2.05 mmol) was dissolved in 37.5 mL of dimethylformamide (DMF), at room temperature. After complete dissolution, triethylamine (TEA, 620 µL, 4.4 mmol) was added and stirred at room temperature for 10 min, followed by the addition of diphenylphosphoryl azide (DPPA, 620 µL, 2.9 mmol). After stirring at room temperature for 30 min, tert-butanol (t-BuOH, 90 mL) was added. Gradual heating (20 °C/h) was carried out up to 90 °C and the reaction was refluxed for 24 h. After purification by semi-preparative RP-HPLC and drying by rotavapor (Büchi Labortechnik AG, Flawil, Switzerland), NBA-BOC was obtained as a mixture of optical isomers and in the form of a flaky solid (Table S1, Figures S1–S4). FTIR (KBr, cm^−1^): 3535, 3296, 3080 (NH), 2979, 2931, 2866 (CH_3_ and CH_2_), 1694 (C=O urethane and C=O biotin). ^1^H NMR (300 MHz, DMSO-d6), δ (ppm): 8.20 (1H, m, NH-1), 7.50 and 7.15 (1H, bs, NH), 6.35 and 6.00 (1H, bs, NH urethane), 4.50 (1H, m, H-3), 4.34 (1H, m, H-4), 3.13 (1H, m, H-5), 2.99 (2H, m, H-7), 2.90 (1H, dd, *J* = 5 Hz, H-6), 2.73 (1H, d, *J* = 12.7 Hz, H-6), 1.70–1.52 (6H, m, H-8,H-9,H-10), 1.45 (9H, s, H-11); ^13^C-NMR (75.5 MHz, CDCl_3_): δ 164.9, 157.6, 79.1, 61.6, 60.5, 55.3, 40.7, 40.4, 29.7, 28.5, 28.3, 25.6.

Compound **1** was hydrolyzed to norbiotinamine (NBA, compound **2**) with TFA:dichloromethane 1:1, at 0 °C for 2 h. Solvents were removed with rotavapor and the obtained product was utilized for the next reaction without further purification.

N-hydroxysuccinimidyl-3-maleimido propionic acid (ASAM) (**3**). This product was prepared as described by Song et al. [17]. β-Alanine (22.4 mmol) and a solution of maleic anhydride (22.4 mmol) in 25 mL of DMF were mixed and stirred at room temperature up to complete dissolution. The resulting solution was cooled to 0 °C in an ice bath, then N-hydroxysuccinimide (28 mmol) was added, followed by *N–N’*-dicyclohexylcarbodiimide (47.7 mmol). The mixture was stirred overnight at room temperature. The precipitated *N,N’*-dicyclohexylurea, formed during the reaction, was removed by centrifugation at 2170 rpm for 10 min, followed by paper filtration. The filtrate was poured in ice to obtain the product as a white precipitate. After centrifugation at 2170 rpm for 10 min and Buchner filtration, the solid was washed with water and dried under vacuum. The yield obtained was 54% *w*/*w* (Table S2, Figures S5–S8). FTIR (KBr, cm^−1^): 3460, 3329 (NH), 3166, 3018 (HC=CH), 2929, 2851 (CH_2_), 1721 (C=O). ^1^H NMR (300 MHz, DMSO-d6), δ (ppm): 2.83 (4H, s, –CH_2_–CH_2_–), 3.02 (2H, t, *J* = 7 Hz, –CH_2_–N–), 3.93 (2H, t, *J* = 7 Hz, –CH_2_–COO–), 6.74 (2H, s, –CH=CH–); ^13^C-NMR (75.5 MHz, CDCl_3_): 25.6 (–CH_2_–CH_2_–), 29.7 (–CH_2_–COO–), 33.0 (–CH_2_–N–), 134.3 (–CH=CH–), 166.0, 168.8, 170.1 (3 –C=O).

N-norbiotinyl-β-maleimidopropionylamide (NAM) (**4**). Compound **2** (about 0.15 mmol) and TEA (0.15 mmol) were dissolved in 25 mL of acetonitrile by sonication. Compound **3** (0.24 mmol) was added to the dispersion, and the reaction mixture was stirred in glycerine bath at 80 °C for 24 h, under reflux. The product was collected by precipitation at −20 °C after 48 h and obtained pure after four washings with diethylether. NAM purity was evaluated by analytical RP-HPLC. The gradient mixture was composed of ultrapure water (solvent A) and acetonitrile gradient grade (solvent B), at 1 mL/min flow rate. Linear gradient from 20% to 100% B in 10 min, with post run to 20% B: 3 min. The chromatograms were acquired at 200, 254 and 340 nm wavelengths. The injection volume was 10 µL and the mobile phase was used as solvent. The obtained yield was 60% *w*/*w* (Table S3, Figures S9–S11). FTIR (KBr, cm^−1^): 3286 (NH), 3091 (HC=CH), 2928, 2856 (CH_2_), 1703 (C=O). ^1^H NMR (300 MHz, DMSO-d6), δ (ppm): 1.10–1.80 (6H, m, –CH_2_–CH_2_–CH_2_–), 2.30 (2H, t, *J* = 7 Hz, –C=O–CH_2_–), 2.70–3.15 (5H, m,–CH_2_–S– + –CH–S– + –CH_2_–NH–), 3.60 (2H, m, –CH_2_–N–), 4.30 (1H, m, –CH–NH– cycle), 4.15 (1H, m, –CH–NH– cycle), 6.40 (2H, dd, –CH=CH–), 7.90 (1H, t, –NH–CH_2_–), NH cycle not detected. The peak attributed to H_2_O is due to the DMSO used for acquisition.

NAMP (**5**). A stock solution of compound **4** was prepared by dissolving 0.6 mg in 1.5 mL of Milli-Q water, in a 1.5 mL polypropylene tube. The peptide solution was prepared by dissolving 1 mg in 500 µL of Milli-Q water. NAM stock solution (1.05 mL, 1.3 µmol) and 450 µL of peptide solution (0.65 µmol) were mixed in a 1.5 mL polypropylene tube, and heated at 37 °C, under orbital shaking at 400 rpm for 24 h, under nitrogen. The product was purified by semi-preparative RP-HPLC. The isocratic mobile phase was composed of 0.1% aqueous TFA and acetonitrile (85:15 *v*/*v*) at 3 mL/min flow rate. The product was characterized by analytical RP-HPLC by applying the same isocratic mobile phase described for semi-preparative RP-HPLC, at 1 mL/min flow rate. Mass spectrometry was performed on an LTQ-Orbitrap Velos Pro mass spectrometer operated in positive ionization mode, at the same conditions described for MacroP mass spectrometry.

### 2.2. Radiochemistry

#### 2.2.1. General Considerations

All chemicals were used as received, without further purification. The water used was purified with a Milli-Q system from Millipore.

#### 2.2.2. Radiolabeling

^68^GaCl_3_ chemical purity. For assessing chemical purity, the concentrations of Fe (at wavelength 259.940 nm) and Zn (at wavelength 213.856 nm) of decayed samples of the ^68^GaCl_3_ solution were measured using atomic emission spectrometry ICP-OES (iCAP 7000 Series, Thermo Scientific, Cambridge, UK) with axial plasma view for better sensitivity. The metal concentrations were lower than the limits of the relative European Pharmacopoeia monograph.

Radiolabeling of MacroP with Gallium-68. Radiolabeling and purification of MacroP were performed with the Eckert & Ziegler Eurotope Modular Lab Pharm-Tracer^®^ automated synthesis system. The ^68^Ge/^68^Ga generator (Eckert & Ziegler GmbH, Berlin, Germany) was eluted with 6 mL of 0.1 M HCl solution and ^68^GaCl_3_ was trapped on a Strata-X-C ion exchange cartridge (SCX, Phenomenex, Castel Maggiore, Italy).

^68^GaCl_3_ was eluted from the cartridge with a mixture composed of 25.0 mL of NaCl 5 M and 62.5 µL of HCl 5.5 M; then 800 µL of the eluted solution were used for the labeling reaction.

The sample solution was prepared by mixing 400 μL of solution A (made by dissolving 0.29 g of sodium acetate in 2 mL of B.Braun water and adding 128 μL of 30% HCl solution) with 2 mL of B.Braun water and 15 μL of 0.833 M MacroP solution. The radiolabeling was performed at 95 °C for 5 min and then some saline, stored at 2–8 °C overnight, was added for cooling. The purification was performed with a C18 ion exchange cartridge (Waters Spa, Sesto San Giovanni, Italy), preconditioned with ethanol/water 1:1 (*v*/*v*) and washed with water. The product was eluted from the cartridge with ethanol/water 1:1 (*v*/*v*) and diluted in saline.

Determination of MacroP radiochemical purity (RCP). The radiochemical purity of MacroP was determined by instant thin-layer chromatography (ITLC) and by RP-HPLC. ITLC was performed on ITLC-SG paper strips and using two different mobile phases: 0.1 M sodium citrate buffer at pH = 5 (Method 1, Figure S12) and 1 M ammonium acetate in water/methanol 1:1 (*v*/*v*) (Method 2, Figure S13). The sample application volumes were 5 μL. The radiochromatographic profile was determined by an autoradiographic system that uses a high-performance storage phosphor screen (Packard BioScience Cyclone CT, Meriden, CT, USA).

RP-HPLC was performed on an UltiMate 3000 UHPLC system (Thermo Fisher Scientific, Waltham, MA, USA) with a Dionex Ultimate 3000 variable wavelength detectors and GABI Star (Raytest), using a Pursuit C18, 3 μm, 3.0 × 150 mm, 200 Å column. For MacroP analysis, a gradient method at a flow rate of 0.6 mL/min was applied with a gradient mixture composed of 0.1% aqueous TFA (solvent A) and acetonitrile gradient grade (solvent B), and gradient elution as follows: 0–2 min 100% B; 2–7 min linear gradient from 100% to 40% B; 7–12 min 40% B; post run to 100% B: 1 min, and 2 min waiting time. The injection volume was 20 µL. The chromatograms were acquired at 220 nm wavelength (Figure S14).

The MacroP complex with native Gallium (^nat^Ga-MacroP) was obtained by the same radiolabeling procedure using Ga(NO_3_)_3_ (Gallium standard solution 1000 µg/mL in 2% nitric acid VWR international Ltd., Leicestershire, England). The product was purified by C18 cartridge and Gallium concentration was determined by ICP-OES (at wavelength 294.364 nm). The purified product was analyzed by RP-HPLC, as described for RCP determination.

Radiolabeling of BisDOTA with Gallium-68. BisDOTA was synthesized according to the procedure described in [18]. BisDOTA radiolabeling was performed with the Eckert & Ziegler Eurotope Modular Lab Standard^®^ automated synthesis system (Eckert & Ziegler GmbH, Berlin, Germany). ^68^Ga was eluted from ^68^Ge/^68^Ga generator with 6 mL of 0.1 M HCl solution and concentrated on a SCX. By rinsing the cation exchange column (Phenomenex, Castel Maggiore, Italy) with 800 μL of 0.02 M HCl in acetone 98%, ^68^GaCl_3_ was eluted into the reaction vial containing 2 mL of 0.2 M sodium acetate buffer (pH = 4) and 7.6 nmol of BisDOTA. The reaction was carried out at 95 °C for 400 s. Without purification, the radiolabeled product was analyzed by ITLC on Whatman MKC18F silica gel plate (60 Å, 2.5 × 7.5 cm, 200 μm layer thickness, Cytiva Europe GmbH, Buccinasco, Italy) without activation. The mobile phase was a mixture of 0.9% NaCl:acetonitrile 1:1 (*v*/*v*), sample application volume of 5 μL. Detection was performed by means of an autoradiographic system using a high-performance storage phosphor screen (Figure S16).

Determination of BisDOTA radiochemical purity (RCP). The radiochemical purity of BisDOTA was determined by RP-HPLC using an UltiMate 3000 UHPLC system with Dionex UltiMate 3000 variable wavelength detectors and GABI Star (Raytest), with a Pursuit C18, 3 μm 3.0 × 150 mm, 200 Å column. For BisDOTA analysis, a gradient method at a flow rate of 0.6 mL/min was applied, with gradient mixture composed of 0.1% aqueous TFA (solvent A) and 0.1% TFA in acetonitrile gradient grade (solvent B), and linear gradient from 5% to 30% B in 20 min. The injection volume was 20 μL. The chromatograms were acquired at 220 nm wavelength (Figure S17).

### 2.3. In Vitro Tests

#### 2.3.1. NAMP–Avidin Binding

Avidin was dissolved in ultrapure water (2.5 μg/μL). This solution (100 μL) was mixed with a NAMP solution (approx. 0.17 mM) and heated at 37 °C under 400 rpm orbital shaking for 2 h. The mixture was placed in an ultrafiltration system with a regenerated cellulose membrane (30 kD cut-off), and washed with 250 μL of ultrapure water at 1400 g for 20 min at 15 °C. By inverted spinning at 1000 g for 3 min, the retentate was recovered and analyzed by capillary electrophoresis (Agilent Technologies 7100 Capillary Electrophoresis, Santa Clara, CA, USA). Separations were performed in a bare fused-silica capillary 50 μm (i.d.) × 64.5 cm (L) × 50 cm (L), activated with 1 M aqueous NaOH, 0.1 M aqueous NaOH and washed with ultrapure water. The conditioning and the run were carried out with 50 mM sodium phosphate buffer (pH 2.5), used also for 120 s preconditioning before injection. The gradient voltage was from 0 to 30 kV in 0.2 min.

#### 2.3.2. NAMP–Avidin–BisDOTA Complex Formation

A volume of 15 μL of a 1 mg/mL BisDOTA solution was added to 36 μL of the retentate derived from NAMP–avidin ultrafiltration. The mixture was ultrafiltered and the retentate was analyzed through capillary electrophoresis at the same conditions reported for the NAMP–avidin binding experiment. In addition, the ultrafiltrate was analyzed, to exclude the presence of BisDOTA.

#### 2.3.3. NAMP–Avidin–^68^Ga-BisDOTA Complex Formation

A volume of 12 µL of 0.031 mM NAMP–avidin solution (corresponding to approx. 0.37 nmol) was added to 500 µL of radiolabeled BisDOTA solution (36 MBq). The reaction was performed at room temperature for 20 min. NAMP–avidin–radiolabeled BisDOTA complex was analyzed by ITLC on Whatman MKC18F silica gel plate (60 Å, size 2.5 × 7.5 cm, layer thickness 200 μm) without activation. The analysis was carried out as described for the ITLC analysis of radiolabeled BisDOTA (Figure S18).

#### 2.3.4. Cell Culture

Human Umbilical Vein Endothelial Cells (HUVEC) were obtained from human umbilical cords collected from full-term women immediately after cesarean section at the Gynecology and Obstetrics Department of International Evangelical Hospital in Genoa (Italy), after patients’ informed consent and the approval (register number 2/2010-19, February, 2010) by the Institutional Ethical Committee. Each cord vein was individually processed for obtaining endothelial cells through mechanical dissociation of the tissue and collagenase digestion. Cells were maintained in culture medium EndoGRO-LS Complete Culture Media Kit seeded on attachment factor-coated plates. For labeling experiments, 8 × 10^5^ cells were plated on Petri dishes in EndoGRO Basal Medium (SCME-BM) and allowed to attach overnight prior stimulation with 10 ng/mL of TNF-alpha (TNF-α) for 4 h. Cells without TNF-α treatment were used as control (Figures S19 and S20).

#### 2.3.5. In Vitro Test of ^68^Ga-MacroP on HUVEC

The binding assay of ^68^Ga-MacroP with VCAM-1 expressed on HUVEC was monitored using LigandTracer^®^ White (Ridgeview Instruments AB, Uppsala, Sweden) according to our previously validated procedure [19]. Briefly, the tool consists of a beta-emission detector and a rotating platform harboring a standard Petri dish. The rotation axis is inclined at 30° from the vertical, so that the cell culture alternates its position from the nadir (for incubation) to the zenith (for counting) every minute. Time–activity curves are thus obtained by subtracting decay-corrected background counting rate from the corresponding target value. Before starting the assay, the culture medium was replaced with 3 mL of the saline solution containing 5 MBq of ^68^Ga-MacroP (specific activity 22.3 MBq/nmol). The binding of radioactivity to cell-containing areas and reference areas was recorded. The same procedures were also applied to a control dish seeded with unstimulated HUVEC.

#### 2.3.6. In Vitro Test of NAMP–avidin–^68^Ga-BisDOTA on HUVEC

After removing the culture medium containing TNF-α, stimulated HUVEC were incubated at 37 °C with 4 nmol of NAMP for 20 min and afterwards with 3.5 nmol of NeutrAvidin™ for 10 min. After washing and replacing the culture medium with 3 mL of the saline solution containing 5MBq of ^68^Ga-BisDOTA (specific activity 37.0 MBq/nmol), the binding assay of ^68^Ga-BisDOTA with the NAMP-avidin complex, bound to the VCAM-1 expressed on HUVEC, was monitored using the same procedure described in Section 2.3.5.

## 3. Results

### 3.1. Synthesis and Characterization of MacroP and NAMP

#### 3.1.1. Synthesis and Characterization of MacroP

MacroP was synthesized by reacting an excess of MMA-DOTA with the VCAM-1-binding peptide in water (Figure 2).

**Figure 4:**
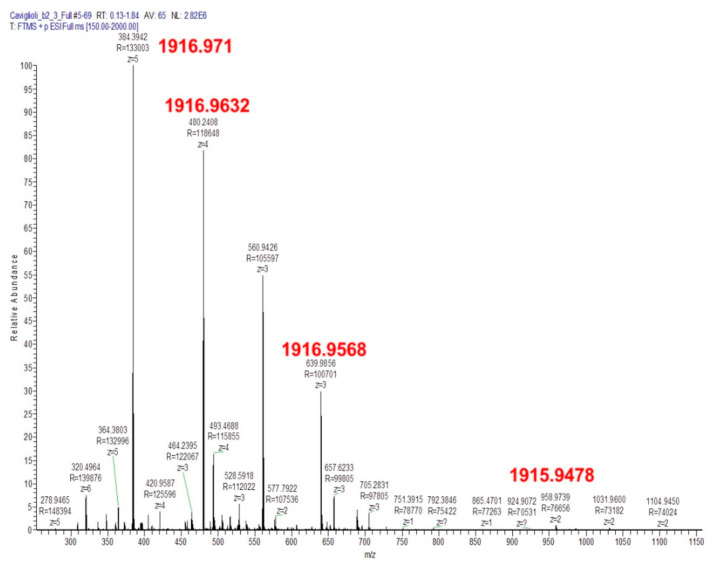
Mass spectrum of MacroP (C_79_H_129_N_29_O_25_S; MW: 1917.114; monoisotopic mass, M_mi_: 1915.943): *m/z* calculated [M+2H]^2+^ 957.9717, [M+3H]^3+^ 638.6478, [M+4H]^4+^ 478.9859, [M+5H]^5+^ 383.1887, [M+6H]^6+^ 319.3239; found [M+2H]^2+^ 958.9739, [M+3H]^3+^ 639.9856, [M+4H]^4+^ 480.2408, [M+5H]^5+^ 384.3942, [M+6H]^6+^ 320.4964.

The resulting product, purified by RP-HPLC, was characterized by mass spectrometry (Figure 4). The calculated monoisotopic mass is 1915.9. The presence of a peak with a mass of 1916.9 is due to the presence of different isotopes of the elements in the molecule.

**Figure 5:**
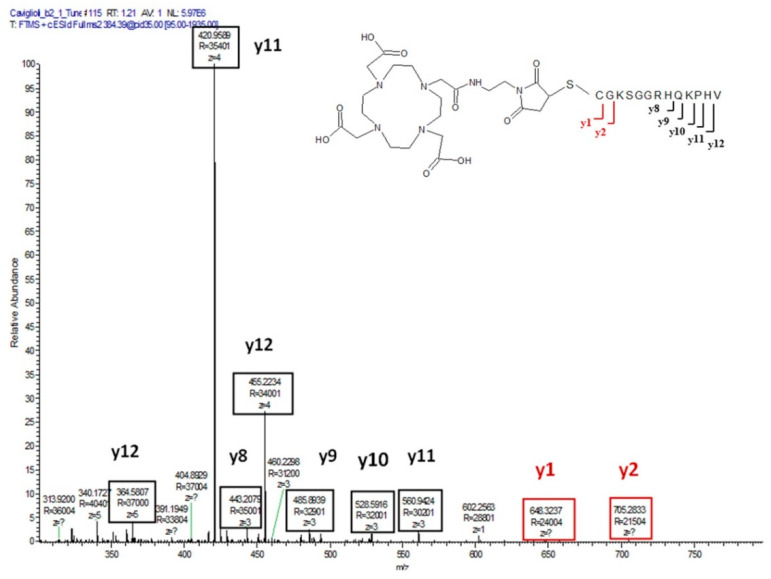
MS^2^ fragmentation spectrum of the peak with *m*/*z* [M+5H]^5+^ of 384.39.

The MS^2^ fragmentation spectrum of the peak with *m*/*z* = 384.39 allowed for the identification of the fragments formed by the cleavage of peptide amide bond (Figure 5). The y1 and y2 ions are related to the fragments of MMA-DOTA conjugated to the cysteine and the cysteine–glycine residues, respectively, thus confirming the conjugation of the peptide through the cysteine and not to the N-terminal valine.

#### 3.1.2. Synthesis and Characterization of NAMP and Precursors

*N-(tert-butoxycarbonyl)-norbiotinamine (NBA-BOC)* (**1**). To synthesize the biotin derivative NAMP, biotin was converted into norbiotinamine through a reaction involving a Curtius rearrangement. Most work in the literature report the norbiotinamine synthesis referring to Szalecki et al. [20], consisting of a one-pot reaction wherein biotin is added to TEA and DPPA in t-BuOH at reflux for 18 h. However, by performing the reaction under the reported conditions, low reaction yield and purity were obtained. Therefore, the mechanism of reaction was studied and, since biotin is only slightly soluble in t-BuOH, the solvent was replaced with DMF. Then, TEA was added and, after 10 min, DPPA. However, considering that t-BuOH, besides being a solvent, is also a reagent, we decided to add it to the reaction mixture composed of biotin, DMF and DPPA after gradual heating to 90 °C. NBA-BOC was purified through semipreparative RP-HPLC and, after vacuum drying, was obtained as a flaky solid (yield of 50%). The product was characterized through elemental analysis, thermal analysis, IR analysis, ^1^H-NMR and ^13^C-NMR (Table S1 and Figures S1–S4).

NBA (**2**) was obtained from hydrolysis of NBA-BOC, using TFA:dichloromethane 1:1. After vacuum drying, the obtained product was used for the next reaction without further purification.

*N-hydroxysuccinimidyl-3-maleimido propionic acid (ASAM)* (**3**). The linker ASAM was obtained following the procedure described by Song et al. [17] (yield of 54%). ASAM was characterized by elemental analysis, DSC, IR analysis, ^1^H-NMR and ^13^C-NMR (Table S2 and Figures S5–S8).

N-norbiotinyl-β-maleimidopropionylamide (NAM) (**4**). NAM synthesis is reported by Szalecki et al. [20]: chloroform was used as solvent and the reaction mixture was kept at room temperature for 18 h. Initially, the reaction was carried out under these conditions, adding TEA (TEA:NBA 1:1 molar ratio) to favor the nucleophilic attack of the amino group of NBA to the activated carboxylic group of ASAM. However, to improve the yield, the reaction conditions were modified by using acetonitrile as a reaction solvent, replacing chloroform. After several attempts, the best yield (60%) was obtained by heating at 80 °C for 24 h. NAM was characterized by elemental analysis, IR analysis and ^1^H-NMR (Table S3 and Figures S9–S11).

*NAMP* (**5**). Finally, NAMP was synthesized by reacting an excess of NAM with the VCAM-1-binding peptide in water.

**Figure 6:**
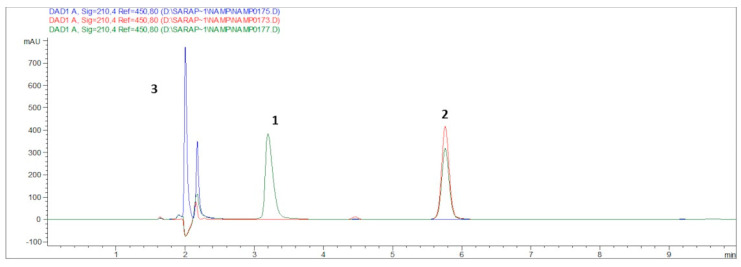
RP-HPLC chromatogram. The green plot is the reaction mixture (reaction time: 24 h) to obtain NAMP; the red plot is purified NAM in water; the blue plot is a VCAM-binding peptide solution in water. Peak 1 (t_R_ = 3.2 min) is related to NAMP, peak 2 (t_R_ = 5.8 min) to NAM and peak 3 (t_R_ = 2.0 min) to the peptide.

The reaction mixture was analyzed by analytical RP-HPLC (Figure 6). The formation of NAMP was evidenced by the appearance of a new peak, spectroscopically pure, at a different retention time. The disappearance of the peptide peak (number 1 in Figure 6) and the decrease in NAM peak (number 2) also confirmed the attribution of peak 3 to NAMP.

**Figure 7:**
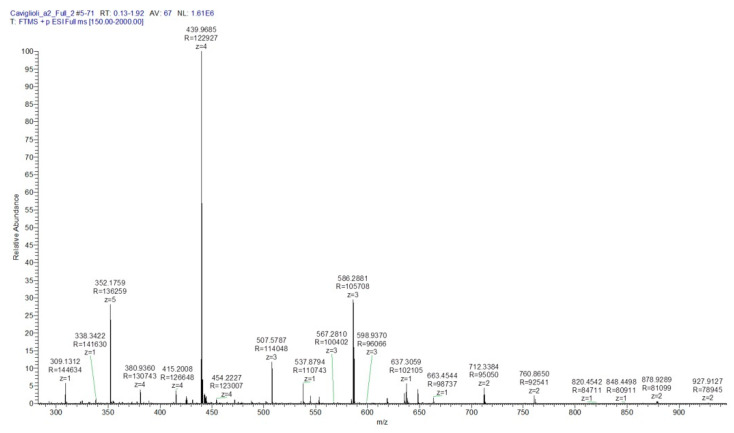
Mass spectrum of NAMP (C_73_H_117_N_27_O_20_S2; MW: 1757.009; M_mi_: 1755.841): *m*/*z* calculated [M+2H]^2+^ 877.9205, [M+3H]^3+^ 585.2803, [M+4H]^4+^ 438.9602, [M+5H]^5+^ 351.1682; found [M+2H]^2+^ 878.9289, [M+3H]^3+^ 586.2881, [M+4H]^4+^ 439.9685, [M+5H]^5+^ 352.1759.

After RP-HPLC purification, the chromatographic band corresponding to peak 1 was isolated and characterized by mass spectrometry, which confirmed the structure of the new radiopharmaceutical (Figure 7), as we have previously demonstrated [21].

**Figure 8:**
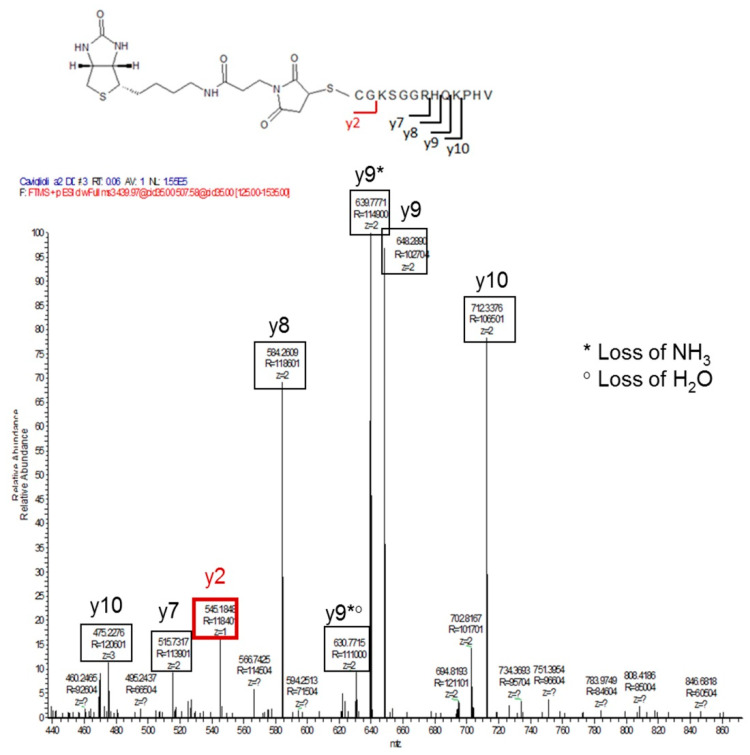
MS^3^ fragmentation spectrum of the peak with *m*/*z* [M+3H]^3+^of 507.5787 [21].

Moreover, the fragmentation in MS^2^ of the peak with *m*/*z* = 439.97 and the fragmentation in MS^3^ of the peak with *m*/*z* = 507.58 allowed for the identification of the fragment ions produced by the progressive cleavage of the peptide amide bond (Figure 8). The y2 ion, in particular, allowed for confirmation that the peptide is conjugated to the maleimide ring by the thiol group of the carboxy-terminal cysteine, and not through the amino group of the amino-terminal valine.

### 3.2. Radiochemistry

#### 3.2.1. Radiolabeling of MacroP

MacroP was radiolabeled with ^68^Ga with a yield of 57%. Radiochemical purity was 97% by ITLC (with both Methods 1 and 2) and 99% by RP-HPLC (Figures S12–S14).

The ^nat^Ga-MacroP complex was synthesized and characterized by ICP-OES and used to identify the radioactive ^68^Ga-MacroP by RP-HPLC comparison. Figure S15 shows the overlaid chromatograms of the mixture reaction with the peak of MacroP and of the solution after reaction time, with the appearance of a new peak corresponding to the same retention time observed in ^68^Ga-MacroP synthesis.

MacroP thermal stability at the labeling conditions (95 °C × 5 min) was evaluated by RP-HPLC. The radiolytic stability of ^68^Ga-MacroP in saline at room temperature was evaluated by ITLC method for 4 h after labeling.

#### 3.2.2. Radiolabeling of BisDOTA

BisDOTA was radiolabeled with ^68^Ga with a yield of 65%. Radiochemical purity of the product, assayed by ITLC (Figure S16) and RP-HPLC (Figure S17), was >90%. In the ITLC chromatogram, it is possible to observe two spots: the weak signal at the deposition line is attributable to trace of ^68^Ga as colloidal impurity, while the second spot corresponds to the migration of the two Bis-DOTA complexes, respectively, containing one and two radionuclides. The two complexes were separated by RP-HPLC [18], as shown in Figure S17.

By ITLC, it has been possible to show the NAMP–avidin–^68^Ga–BisDOTA complex formation. In fact, in Figure S18 it is possible to appreciate that the spots are two: one is relative to NAMP–avidin–^68^Ga–BisDOTA, while the second spot can be attributed to an excess of ^68^Ga–BisDOTA that has not bound the protein.

### 3.3. In Vitro Tests

#### 3.3.1. NAMP–avidin and NAMP–avidin–BisDOTA Complex Formation

**Figure 9:**
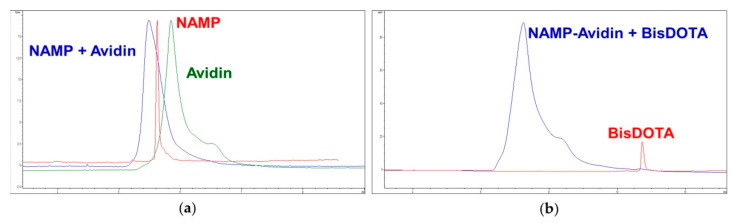
Capillary electrophoresis analysis. (**a**) Comparison among NAMP (red), avidin (green) and the retentate resulting from the ultrafiltration of a NAMP–avidin mixture (blue). (**b**) Comparison between BisDOTA (red) and the retentate resulting from the ultrafiltration of a NAMP–avidin–BisDOTA mixture (blue).

An ultrafiltration test demonstrated the NAMP-avidin complex formation. In fact, no unbound NAMP could be detected in the ultrafiltrate by RP-HPLC method. By capillary electrophoresis, it was possible to highlight the presence of NAMP–avidin complex in the retentate (Figure 9a), ruling out the non-specific adsorption of NAMP to the filter.

Then, to verify the formation of the NAMP–avidin–BisDOTA complex, a BisDOTA solution was added to the retentate of NAMP–avidin ultrafiltration. The capillary electrophoresis analysis of the retentate evidenced the formation of the complex and the disappearance of the BisDOTA peak (Figure 9b), that was also absent in the filtrate.

The formation of NAMP–avidin–radiolabeled BisDOTA complex was also confirmed by ITLC (Figure S18).

#### 3.3.2. In Vitro Tests on HUVEC

The in vitro cell tests were performed on HUVEC cells activated with TNF-α, in order to induce VCAM-1 expression on cell membranes (Figures S19 and S20).

**Figure 10:**
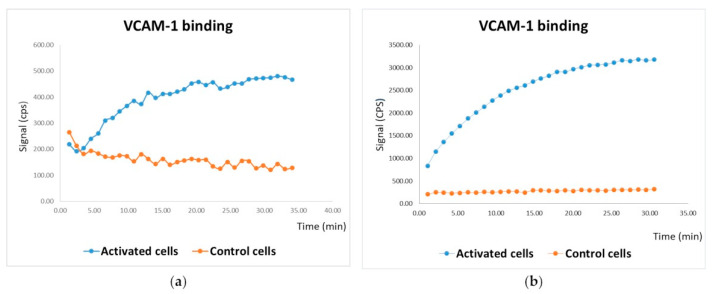
(**a**) In vitro test of MacroP on HUVEC. Control cells (orange) were not activated with TNF-α before being treated with ^68^Ga-MacroP; activated cells (blue) had been previously exposed to TNF-α. (**b**) In vitro test of NAMP on HUVEC. Control cells (orange) were not activated with TNF-α; both activated and control cells received NAMP, followed by NeutAvidin and ^68^Ga–BisDOTA.

By using a Ligandtracer^®^, it was observed that the radioactive uptake of ^68^Ga–MacroP was higher in activated cells compared to the control cells (Figure 10a) (*p* < 0.05). The same result was obtained by treating the cells with NAMP, followed by NeutrAvidin™ and ^68^Ga–BisDOTA. Additionally, in this case, activated cells exhibited an evidently higher uptake of radioactivity compared to the control cells (Figure 10b) (*p* < 0.05).

## 4. Discussion

Vascular Cell Adhesion Molecule-1 plays a crucial role in atherosclerotic plaque progression. This adhesion protein is rapidly upregulated during the first stages of plaque formation; therefore, it is a very attractive imaging biomarker for the early detection of atheroma formation.

Two novel radiopharmaceuticals, based on a VCAM-1 binding peptide, were synthesized and thoroughly characterized.

MacroP has only one DOTA ring linked to the VCAM-1 binding peptide; therefore, it can chelate just one radionuclide, as with the most common diagnostics currently in use.

In order to further increase the diagnostic sensitivity, a pretargeting strategy, including NAMP with the use of a double chelating molecule (BisDOTA), was conceived. However, although BisDOTA can theoretically bind up to two radionuclide ions, as shown in Figure S17, at the labeling conditions here described, this happens for an undetermined fraction of the labeled BisDOTA molecules, with the remaining molecules complexing only one radionuclide. This issue has been raised by other researchers [22,23,24] and Storch et al. [24] concluded that BisDOTA derivatives showed improved specific activity and labeling kinetics.

Another drawback possibly occurring in protein-based radiolabeled tracers, as MacroP, is due to the labeling conditions, which could damage the peptide moiety, decreasing or eliminating the affinity for target receptor. In this case, the stability of MacroP was tested during a simulated labeling process (heating for 5 min at 95 °C) allowing for the exclusion of any thermal degradation of the molecule. Moreover, the stability of ^68^Ga–MacroP in saline was confirmed by ITLC analysis for at least 4 h at room temperature, also excluding degradation by radiolysis (Table S4).

Before performing the reactions involving the VCAM-1-binding peptide, the possibility of the formation of dipeptide molecules due to disulfuric bridges was considered, and the introduction of the reducing agent tris-(2-carboxyethyl)phosphine hydrochloride (TCEP) in the reaction mixtures was evaluated. Interestingly, when the peptide was dissolved in water, no dipeptide was detected by RP-HPLC analysis, while the dipeptide formation was observed in phosphate buffer (Figures S21 and S22). Considering that it has been recently reported that TCEP might lead to the formation of secondary products [25,26,27,28], its use was avoided, and we decided to perform the reactions involving the peptide in pure water.

The characterization of MacroP and NAMP by mass spectroscopy is a key point because the fragmentation spectra of the compounds allowed for the confirmation of the correct conjugation of the peptide to the maleimide ring through the C-terminal cysteine and not the N-terminal valine. Indeed, even if the reaction with the sulfhydryl group is favored at neutral pH, the amino group of valine might also react. The valine residue seems to be fundamental for the binding to VCAM-1, so it has to be free to interact with the target [12].

NAMP, a biotin derivative of the VCAM-1 binding peptide, was conceived to be used in a two- or three-step pretargeting procedure, based on the avidin/biotin high-affinity complex [15]. The in vitro study reported here regards the three-step approach, where NAMP, avidin and the radiolabeled molecule (BisDOTA) were sequentially applied [13,14].

Before performing the in vitro tests on cells, we confirmed that the biotin moiety of NAMP retained its avidin binding capacity, which might be hampered by the modification of the biotin structure. Moreover, the formation of the NAMP-avidin-BisDOTA complex was also verified to confirm that the presence of a NAMP molecule bound to avidin did not prevent its binding to the BisDOTA biotin moiety.

After radiolabeling with ^68^Ga, both MacroP and NAMP were tested on human endothelial cells. Quantitative PCR and Fluorescence-Activated Cell Sorting (FACS) analysis were performed prior to the experiments, to assess the expression of VCAM-1 on HUVEC. To avoid excessive stress for the cells, they were activated with TNF-α for 4 h only. In the three-step pretargeting test, a commercially available deglycosylated form of avidin (NeutrAvidin™) was employed because it is characterized by reduced non-specific binding, low isoelectric point (~6.3) and low production cost [29,30] with respect to avidin.

Both radiopharmaceuticals exhibited VCAM-1 binding capacity. In the case of MacroP, activated cells showed 4-fold higher radiosignal uptake compared to control cells, while with NAMP the radioactive uptake of activated cells was 12-fold higher compared to the controls. For both compounds, the time–activity curve was compatible with accumulation kinetics suggesting an irreversible link between the tracer and its receptor. The procedure involving NAMP gave the higher radiosignal deriving from VCAM-1 expressing cells because of the use of BisDOTA, which, being able to chelate either one or two radioisotopes per molecule, increases the signal deriving from the target. In fact, using the same number of moles in the labeling procedure the specific activity of BisDOTA resulted 1.4–1.7 fold higher than that of MacroP.

Having excluded the stability problems of MacroP, the higher uptake of NAMP could be explained either with higher affinity for VCAM-1 or with higher rate of accumulation. Actually, if the affinity of the two molecules was not different, the higher radiosignal for NAMP procedure could be explained with the higher amount of radioactivity carried by BisDOTA or by avidin ability to complex more than one molecule of BisDOTA. To clarify these aspects, studies of binding with VCAM-1 are in progress for both molecules.

Future tests on atherosclerosis-prone apolipoprotein E-deficient (Apoe−/−) mice are planned to assess the in vivo efficiency of the newly developed PET radiotracers and to investigate their in vivo characteristics.

As MacroP features low molecular weight, it might exhibit short plasma half-life and fast renal clearance, generating a higher signal-to-noise ratio, improving patient safety. Conversely, the short half-life of MacroP might limit its accumulation in the early lesion sites, reducing the radiosignal.

One of the advantages of using NAMP in a two- or three-step pretargeting system consists of a reduced uptake of radioactives by healthy tissues, shorter time to achieve adequate tumor-to-background ratios, and the possibility to employ short-lived radionuclides, such as ^68^Ga. Moreover, the pretargeting strategies usually include one or more chasing steps, effective in reducing background signal.

Even if pretargeting has been initially conceived for imaging purposes, these kinds of strategies are under investigation for their possible application in the therapeutic field. Therefore, the new pretargeting system, based on NAMP, might represent a flexible theranostic tool that is exploitable for both diagnoses, by using a biotinylated radiotracer, and therapy, by using a biotinylated nanocarrier encapsulating an active compound with anti-inflammatory activity. Indeed, a study on the development of a biotinylated nanocarrier encapsulating different anti-inflammatory drugs is currently ongoing [21].

## 5. Conclusions

Two novel VCAM-1-targeting radiopharmaceuticals, based on the VCAM-1-binding peptide, with sequence VHPKQHRGGSKGC, have been successfully synthesized and characterized, and their radiolabeling with ^68^Ga led to high yields and radiochemical purity. Both compounds resulted in the retention of the VCAM-1 binding ability. The potential superiority of NAMP in the labeling procedure, biodistribution and PET sensitivity makes it a promising compound to be used in a two- or three-step pretargeting system for theranostic applications, in association with a biotinylated PET radiotracer or a biotinylated nanocarrier, encapsulating anti-inflammatory activities.

## Supplementary Materials

The following are available online at https://www.mdpi.com/article/10.3390/pharmaceutics13071025/s1, Determination of MacroP chelating efficiency; Table S1: Elemental analysis of NBA-BOC; Figure S1: DSC profile of NBA-BOC; Figure S2: IR spectrum of NBA-BOC; Figure S3: ^1^H-NMR of NBA-BOC; Figure S4: ^13^C-NMR of NBA-BOC; Table S2: Elemental analysis of ASAM; Figure S5: DSC profile of ASAM; Figure 6: IR spectrum of ASAM; Figure S7: ^1^H-NMR of ASAM; Figure S8: ^13^C-NMR of ASAM; Table S3: Elemental analysis of NAM; Figure S9: IR spectrum of NAM; Figure S10: ^1^H-NMR of NAM; Figure S11: RP-HPLC chromatogram of NAM; Figure S12: ITLC of raw ^68^Ga–MacroP with Method 1; Figure S13: ITLC of raw ^68^Ga–MacroP with Method 2; Figure S14: RP-HPLC of pure ^68^Ga–MacroP; Figure S15: RP-HPLC of MacroP simulated labeling; Figure S16: ITLC of ^68^Ga–BisDOTA; Figure S17: RP-HPLC of ^68^Ga–BisDOTA; Figure S18: ITLC of NAMP–avidin–^68^Ga–BisDOTA; Table S4: Stability study of ^68^Ga–MacroP; Figure S19: Q-PCR of TNF-α activated HUVEC; Figure S20: FACS of TNF-α activated HUVEC; Figure S21: RP-HPLC comparison between the peptide dissolved in water and in phosphate buffer; Figure S22: RP-HPLC comparison between the peptide dissolved in phosphate buffer before and after TCEP addition.

## References

[1] Barquera S., Pedroza-Tobias A., Medina C., Hernandez-Barrera L., Bibbins-Domingo K., Lozano R., Moran A.E. (2015). Global overview of the epidemiology of atherosclerotic cardiovascular disease. Arch. Med. Res..

[2] Galkina E., Ley K. (2007). Vascular adhesion molecules in atherosclerosis. Arterioscler. Thromb. Vasc. Biol..

[3] Lusis A.J. (2000). Atherosclerosis. Nature.

[4] Libby P. (2002). Inflammation in atherosclerosis. Arterioscler. Thromb. Vasc. Biol..

[5] Libby P., Ridker P.M., Maseri A. (2002). Inflammation and atherosclerosis. Circulation.

[6] Cybulsky M.I., Gimbrone M.A. (1991). Endothelial expression of a mononuclear leukocyte adhesion molecule during atherogenesis. Science.

[7] Osborn L., Hession C., Tizard R., Vassallo C., Luhowskyj S., Chirosso G., Lobb R. (1989). Direct expression cloning of vascular cell adhesion molecule 1, a cytokine-induced endothelial protein that binds to lymphocytes. Cell.

[8] Cybulsky M.I., Iiyama K., Li H., Zhu S., Chen M., Iiyama M., Davis V., Gutierrez-Ramos J.C., Connelly P.W., Milstone D.S. (2001). A major role for VCAM-1, but not ICAM-1, in early atherosclerosis. J. Clin. Invest..

[9] Ailuno G., Baldassari S., Zuccari G., Schlich M., Caviglioli G. (2020). Peptide-based nanosystems for vascular cell adhesion molecule-1 targeting: A real opportunity for therapeutic and diagnostic agents in inflammation associated disorders. J. Drug Deliv. Sci. Technol..

[10] Ailuno G., Zuccari G., Baldassari S., Lai F., Caviglioli G. (2021). Anti-Vascular Cell Adhesion Molecule-1 nanosystems: A promising strategy against inflammatory based diseases. J. Nanosci. Nanotechnol..

[11] Asati S., Pandey V., Soni V. (2019). RGD peptide as a targeting moiety for theranostic purpose: An update study. Int. J. Pept. Res. Ther..

[12] Kelly K.A., Nahrendorf M., Yu A.M., Reynolds F., Weissleder R. (2006). In vivo phage display selection yields atherosclerotic plaque targeted peptides for imaging. Mol. Imaging Biol..

[13] Altai M., Membreno R., Cook B., Tolmachev V., Zeglis B.M. (2017). Pretargeted Imaging and Therapy. J. Nucl. Med..

[14] Verhoeven M., Seimbille Y., Dalm S.U. (2019). Therapeutic Applications of Pretargeting. Pharmaceutics.

[15] Paganelli G., Chinol M. (2003). Radioimmunotherapy: Is avidin-biotin pretargeting the preferred choice among pretargeting methods?. Eur. J. Nucl. Med. Mol. Imaging.

[16] Caviglioli G., Chinol M., Baldassari S., Garaboldi L., Zuccari G., Petretto A., Drava G., Sinico C., Paganelli G. (2019). A new microdispersed albumin derivative potentially useful for radio-guided surgery of occult breast cancer lesions. Sci. Rep..

[17] Song H.Y., Ngai M.H., Song Z.Y., MarcAry P.A., Hobley J., Lear M.J. (2009). Practical synthesis of maleimides and coumarin-linked probes for protein and antibody labelling via reduction of native disulfides. Org. Biomol. Chem..

[18] Pratesi A., Bucelli F., Mori I., Chinol M., Verdoliva A., Paganelli G., Rivieccio V., Gariboldi L., Ginanneschi M. (2010). Biotin derivatives carrying two chelating DOTA units. Synthesis, in vitro evaluation of biotinidases resistance, avidin binding, and radiolabeling tests. J. Med. Chem..

[19] Cossu V., Marini C., Piccioli P., Rocchi A., Bruno S., Orengo A.M., Emionite L., Bauckneht M., Grillo F., Capitanio S. (2019). Obligatory role of endoplasmic reticulum in brain FDG uptake. Eur. J. Nucl. Med. Mol. Imaging.

[20] Szalecki W. (1996). Synthesis of norbiotinamine and its derivatives. Bioconjug. Chem..

[21] Ailuno G., Baldassari S., Zuccari G., Di Francesco V., Decuzzi P., Caviglioli G. Poster Communication. Proceedings of the NanoInnovation 2020 Conference.

[22] Storch D., Schmitt J.S., Waldherr C., Waser B., Reubi J.C., Maecke H.R. (2007). Preclinical evaluation of somatostatin analogs bearing two macrocyclic chelators for high specific activity labeling with radiometals. Radiochim. Acta.

[23] Song S.L., Xiong C.Y., Zhou M., Lu W., Huang Q., Ku G., Zhao J., Flores L.G., Ni Y.C., Li C. (2011). Small-Animal PET of Tumor Damage Induced by Photothermal Ablation with Cu-64-Bis-DOTA-Hypericin. J. Nucl. Med..

[24] Prinsen K., Cona M.M., Cleynhens J., Vanbilloen H., Li J.J., Dyubankova N., Lescrinier E., Bormans G., Ni Y.C., Verbruggen A. (2013). Synthesis and biological evaluation of ^68^Ga labeled bis-DOTA-3,3′-(benzylidene)-bis-(1H-indole-2-carbohydrazide) as a PET tracer for in vivo visualization of necrosis. Bioorg. Med. Chem. Lett..

[25] Shafer D.E., Inman J.K., Lees A. (2000). Reaction of Tris(2-carboxyethyl)phosphine (TCEP) with maleimide and α-haloacyl groups: Anomalous elution of TCEP by gel filtration. Anal. Biochem..

[26] Tyagarajan K., Pretzer E., Wiktorowicz J.E. (2003). Thiol-reactive dyes for fluorescence labeling of proteomic samples. Electrophoresis.

[27] Nair D.P., Podgórski M., Chatani S., Gong T., Xi W., Fenoli C.R., Bowman C.N. (2014). The thiol-Michael addition click reaction: A powerful and widely used tool in materials chemistry. Chem. Mater..

[28] Kantner T., Watts A.G. (2016). Characterization of reactions between water-soluble trialkylphosphines and thiol alkylating reagents: Implications for protein-conjugation reactions. Bioconj. Chem..

[29] Chinol M., Casalini P., Maggiolo M., Canevari S., Omodeo E.S., Caliceti P., Veronese F.M., Cremonesi M., Chiolerio F., Nardone E. (1998). Biochemical modifications of avidin improve pharmacokinetics and biodistribution, and reduce immunogenicity. Br. J. Cancer.

[30] Jain A., Barve A., Zhao Z., Jin W., Cheng K. (2017). Comparison of avidin, neutravidin, and streptavidin as nanocarriers for efficient siRNA delivery. Mol. Pharm..

