# Supplementary material for: Two Novel PET Radiopharmaceuticals for Endothelial Vascular Cell Adhesion Molecule-1 (VCAM-1) Targeting
Source: Pharmaceutics. 2021 Jul 6;13(7):1025. doi: 10.3390/pharmaceutics13071025 (PMC8309178; doi:10.3390/pharmaceutics13071025)
Supplement: Supplementary file 1 [file pharmaceutics-13-01025-s001.zip › pharmaceutics-1254252-supplementary.pdf]

pharmaceutics

Supplementary Materials: Two Novel PET Radiopharmaceuticals for Endothelial Vascular Cell Adhesion Molecule-1 (VCAM-1) Targeting

Sara Pastorino, Sara Baldassari, Giorgia Ailuno, Guendalina Zuccari, Giuliana Drava, Andrea Petretto, Vanessa Cossu, Cecilia Marini, Silvana Alfei, Tullio Florio, Gianmario Sambuceti and Gabriele Caviglioli

Determination of MacroP chelating efficiency

Since during synthesis and purification of MacroP a metallic contamination could occur, hampering MacroP capacity to chelate the radionuclide, a spectrophotometric assay was carried out to evaluate MacroP chemical purity [16]. The assay is based on the decrease of absorption at 656 nm of the complex Pb^{2+}-arsenazo (AA) due to the AA displacement and formation of Pb^{2+}-DOTA complex.

A stock solution of Pb^{2+}-AA in ammonium acetate buffer (0.15 M, pH = 7) containing 67.62 μmol/L of Pb^{2+} and 140 μmol/L of AA was prepared; DOTA was dissolved in the same ammonium acetate buffer (0.26 mM). For the calibration curve, standard solutions containing increasing concentrations of DOTA (0-0.024 mM) were prepared by mixing 3.4 mL of Pb^{2+}-AA stock solution, 200 μL of 1 M NaCl (in 0.15 M ammonium acetate buffer) and a variable volume of DOTA stock solution (0-500 μL). The final 4.2 mL volume of each standard solution was reached by adding a suitable volume of ammonium acetate buffer.

The sample solution was prepared by adding 40 μL of a 1.6 mM MacroP aqueous solution to a mixture containing 3.4 mL of Pb^{2+}-AA stock solution, 200 μL of 1 M NaCl and 560 μL of ammonium acetate buffer. For each solution, absorbance at 656 nm was determined, 10 min after its preparation, at room temperature and protected from light. The reading was corrected by subtracting the absorbance of a mixture made of 4.0 mL of ammonium acetate buffer and 200 μL of 1 M NaCl.

Table 1. Elemental analysis of NBA-BOC.

	%C	%H	%N	%S
**Theoretical**	53.31	7.99	13.32	10.16
**Actual**	53.35	8.13	13.62	10.04

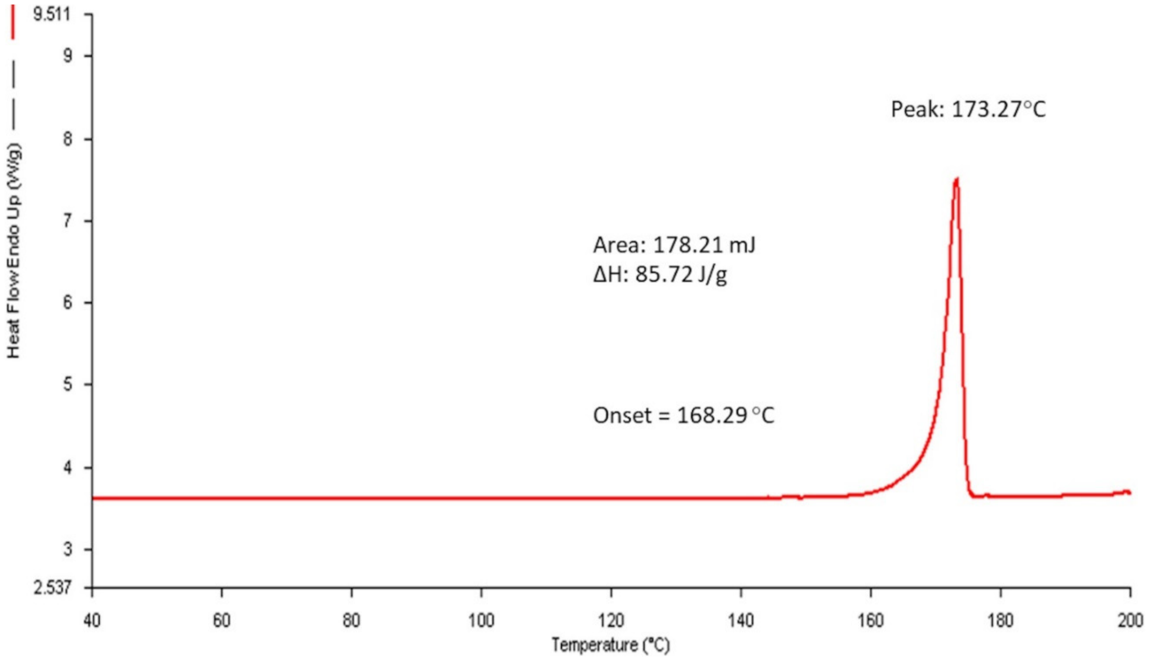

Figure S1. DSC profile of NBA-BOC heated from 40 °C to 200 °C at 10 °C/min.

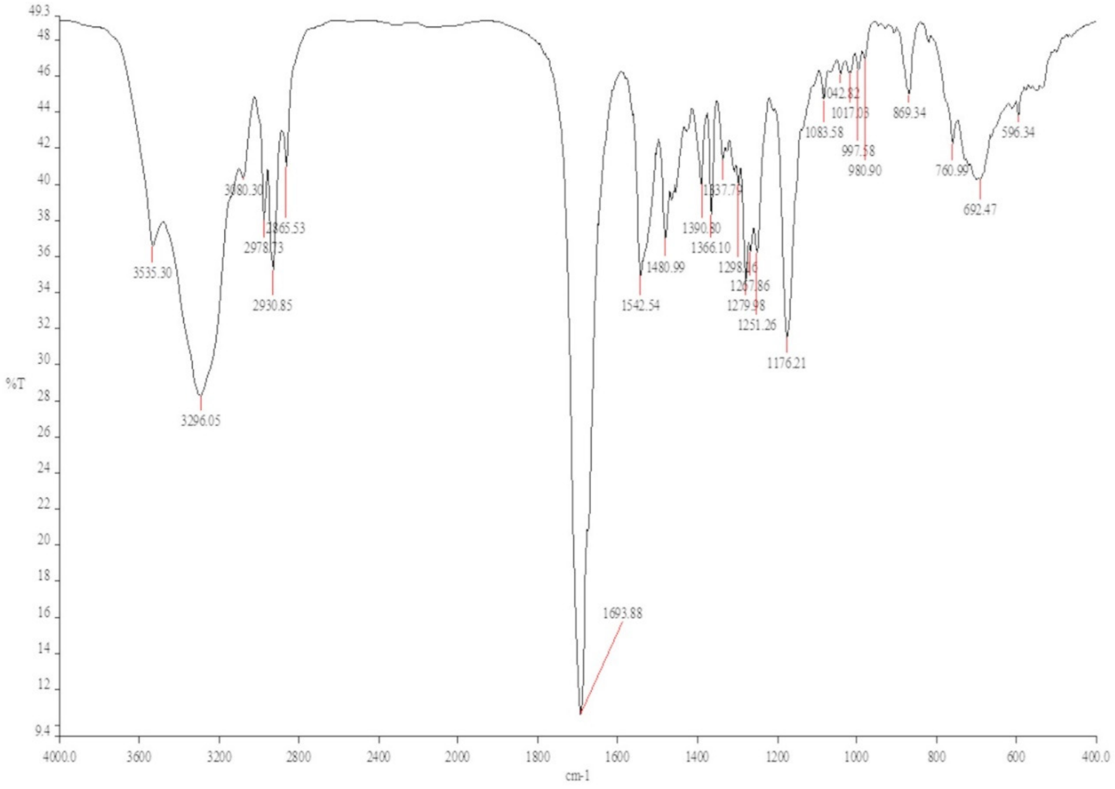

Figure S2. IR spectrum of NBA-BOC.

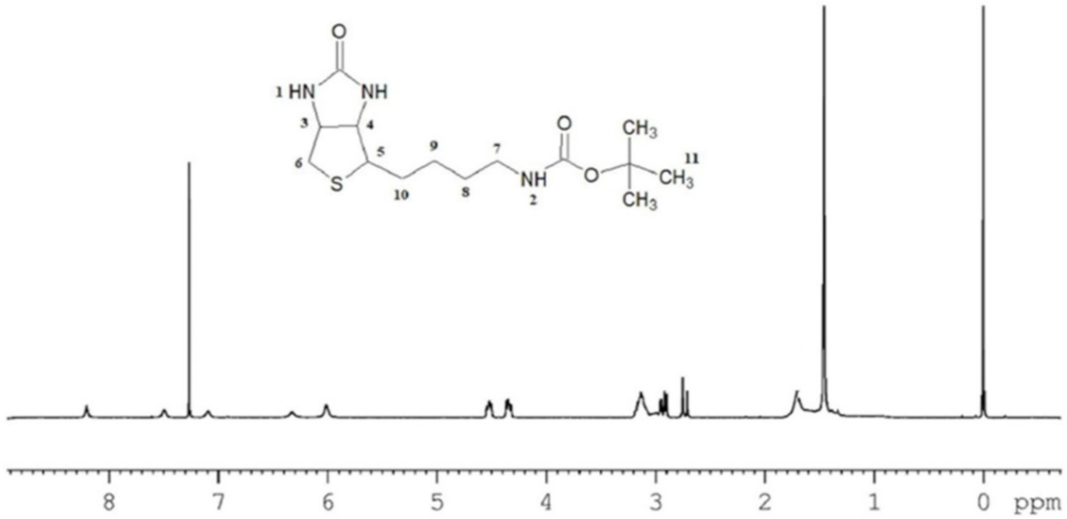

Figure S3. ^{1}H-NMR of NBA-BOC in $CDCl_3$.

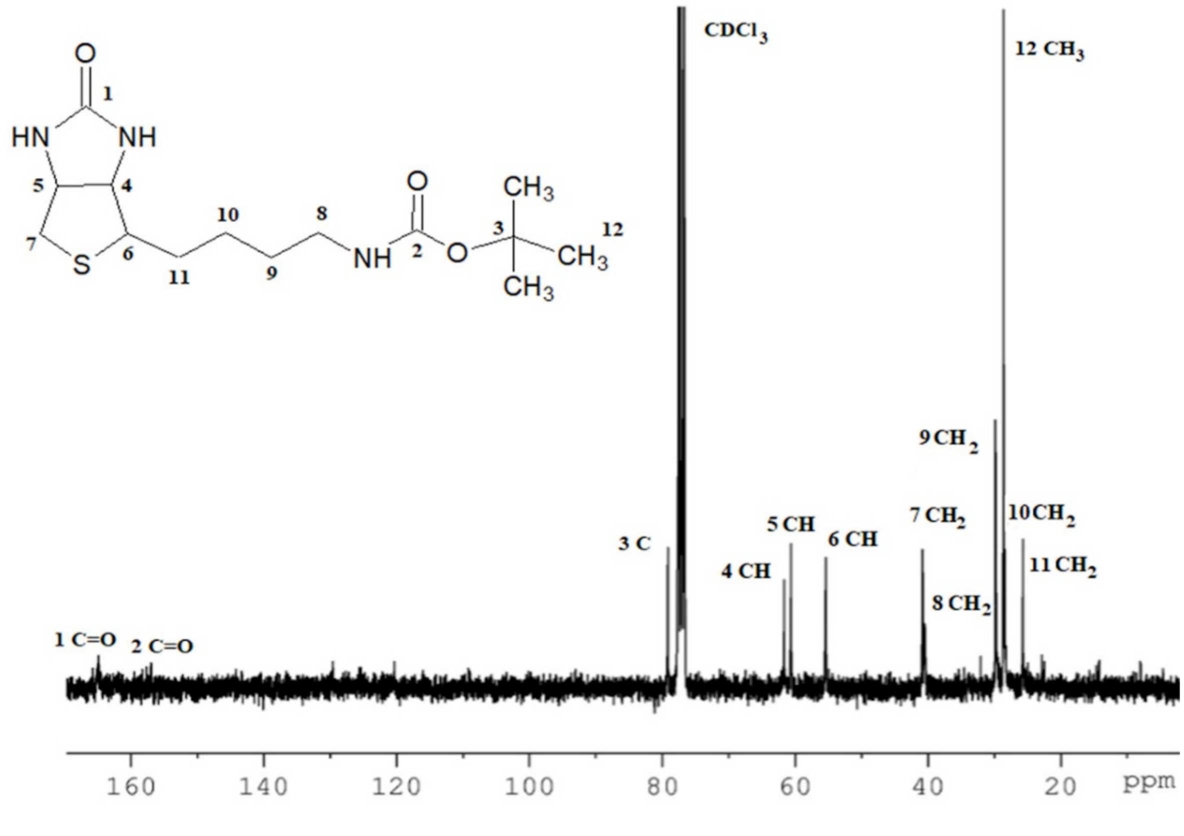

Figure S4. ^{13}C-NMR of NBA-BOC in $CDCl_3$.

Table S2. Elemental analysis of ASAM.

	%C	%H	%N
Theoretical	49.63	3.79	10.52
Actual	49.45	3.80	10.35

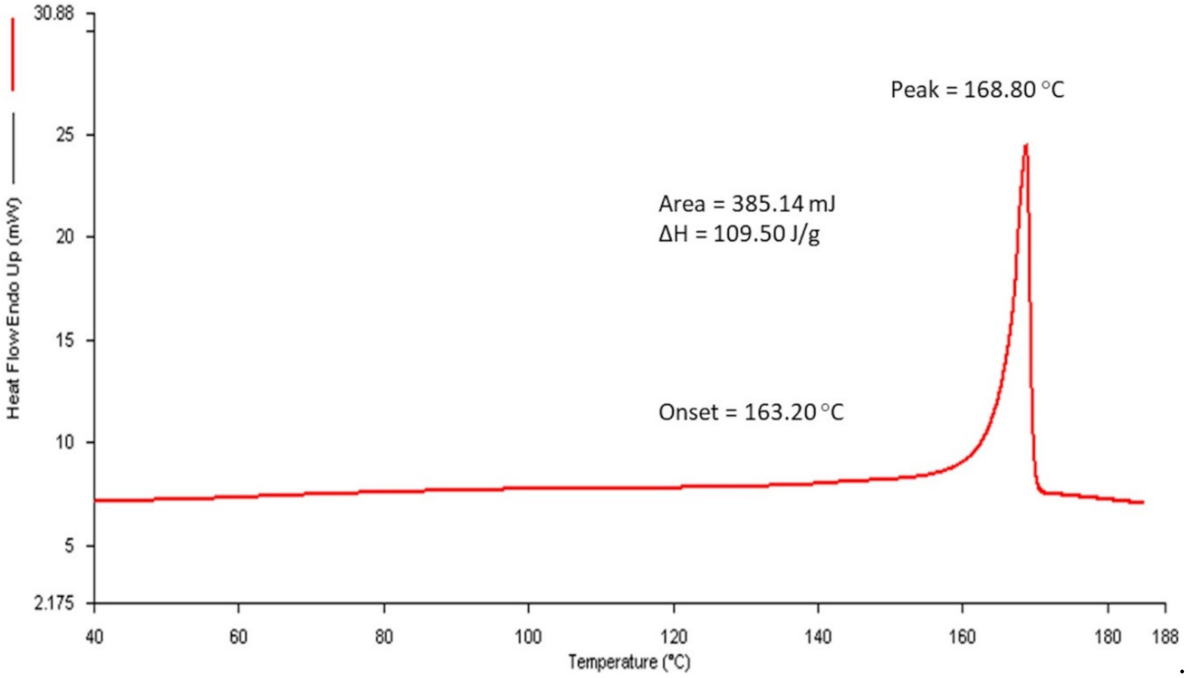

Figure S5. DSC profile of ASAM heated from 30 °C to 185 °C at 10 °C/min.

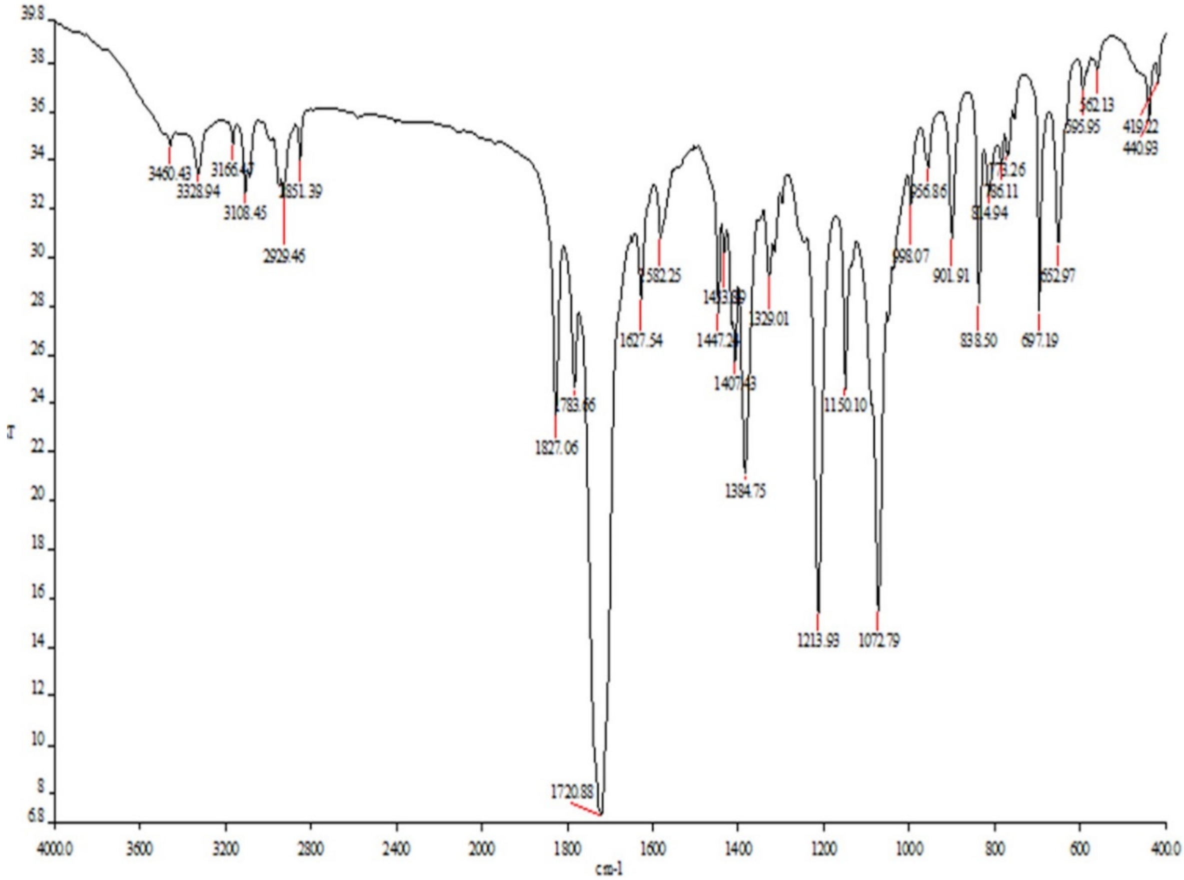

Figure S6. IR spectrum of ASAM.

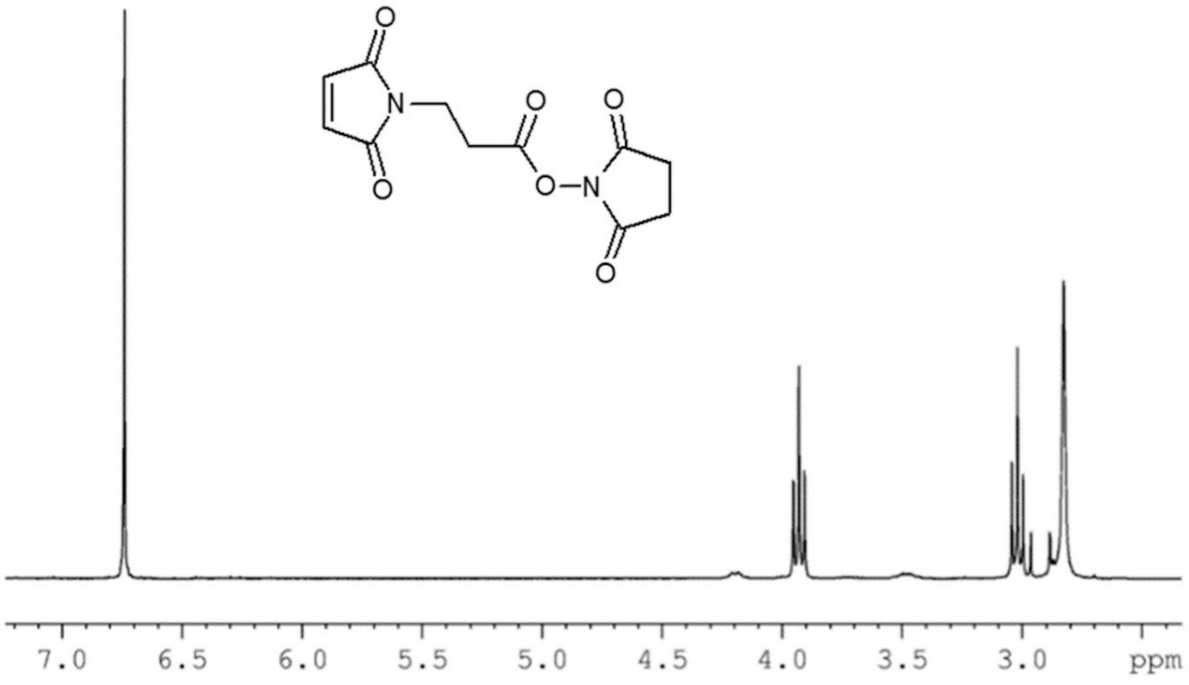

Figure S7. ^{1}H-NMR of ASAM.

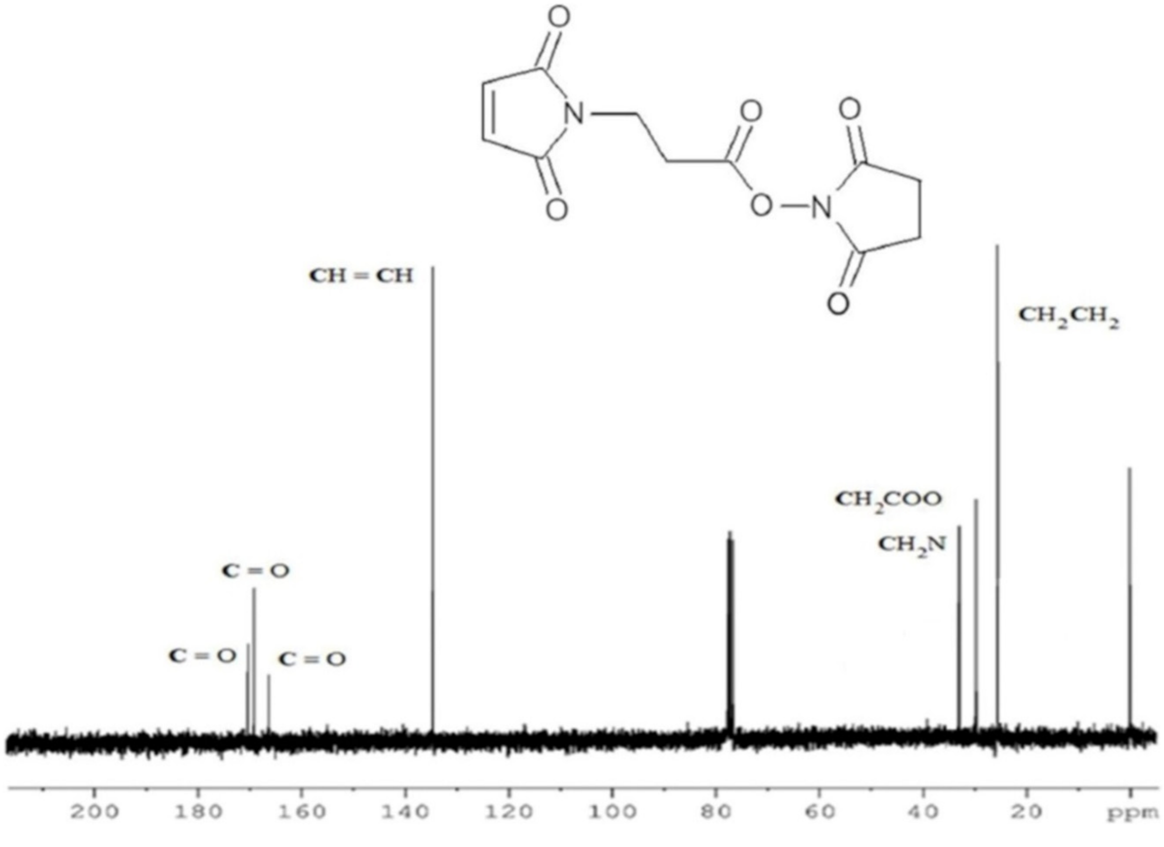

Figure S8. ^{13}C-NMR of ASAM.

Table S3. Elemental analysis of NAM.

	%C	%H	%N	%S
**Theoretical**	52.45	6.05	15.29	8.75
**Actual**	52.46	6.09	14.98	9.50

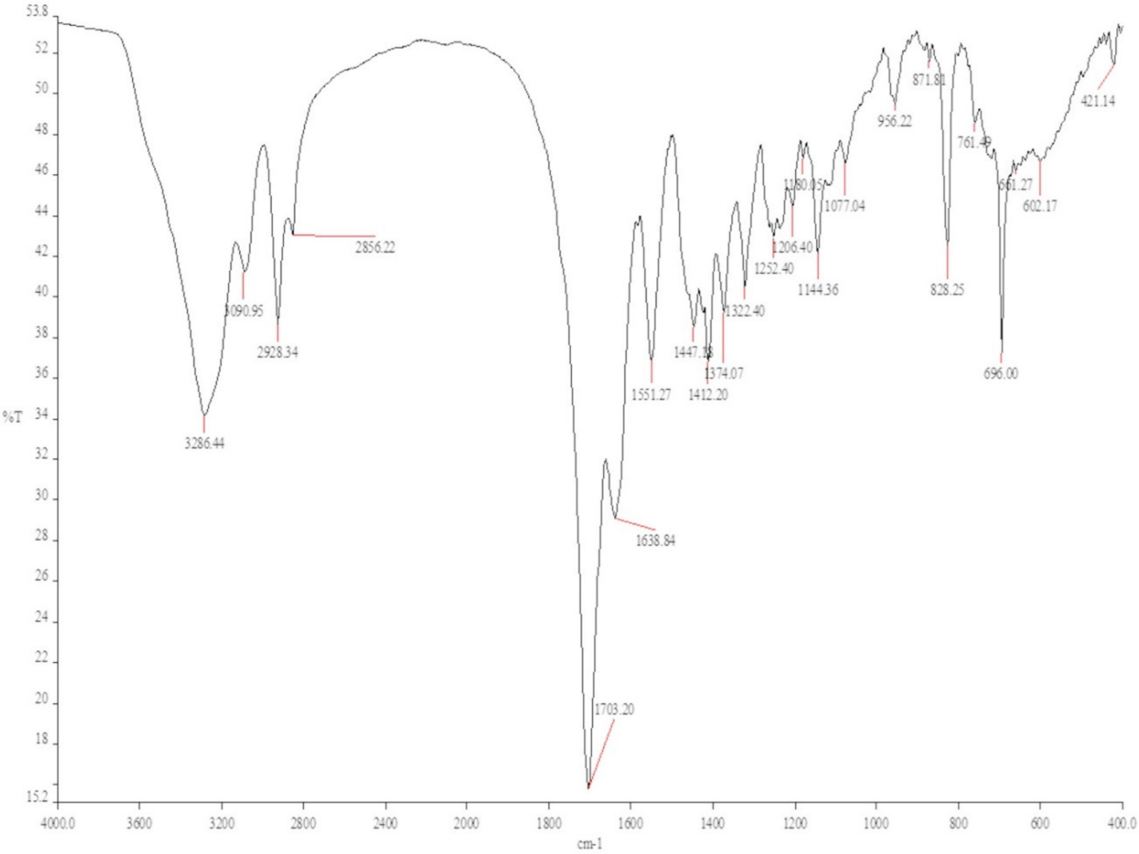

Figure S9. IR spectrum of NAM.

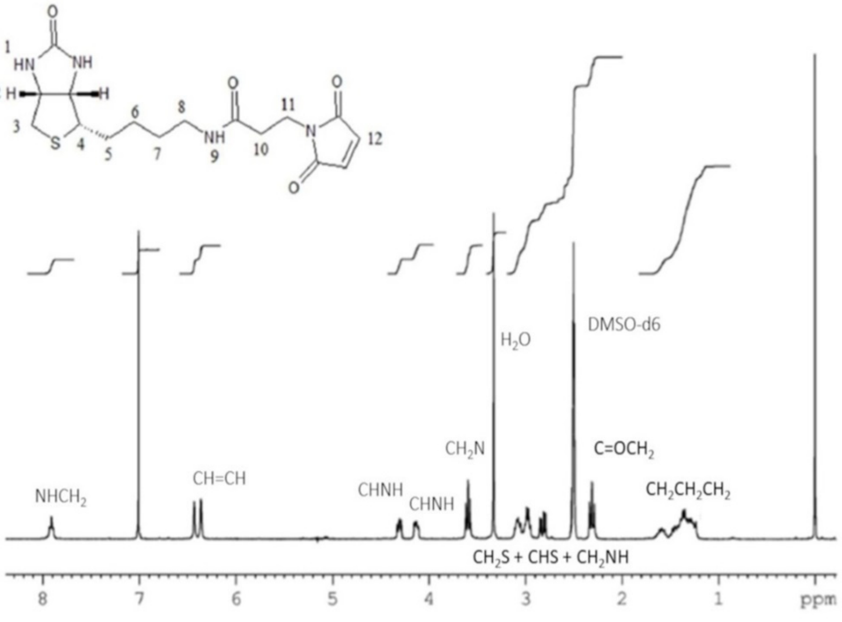

Figure S10. ^{1}H-NMR of NAM.

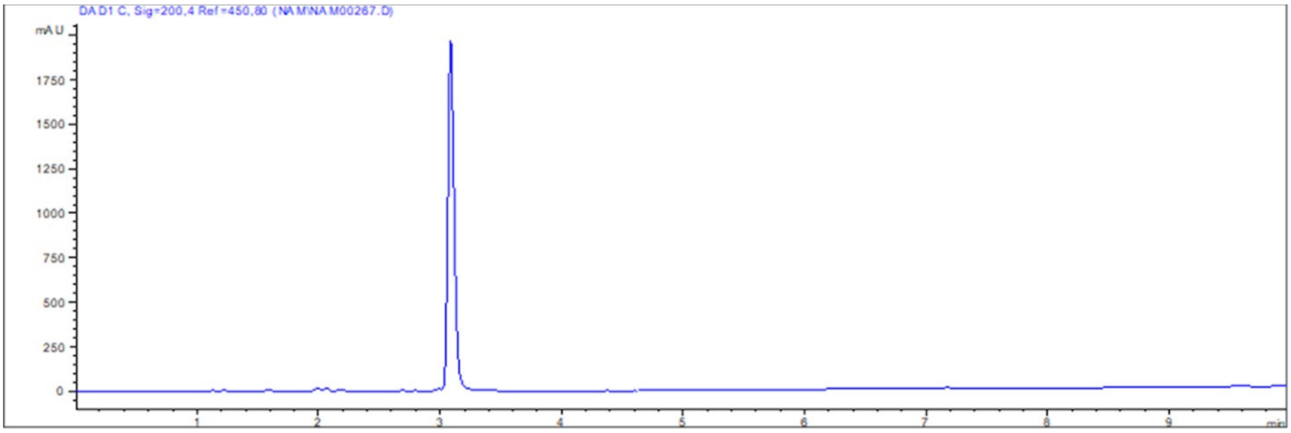

Figure S11. RP-HPLC chromatogram of NAM. Detection wavelength: 200 nm.

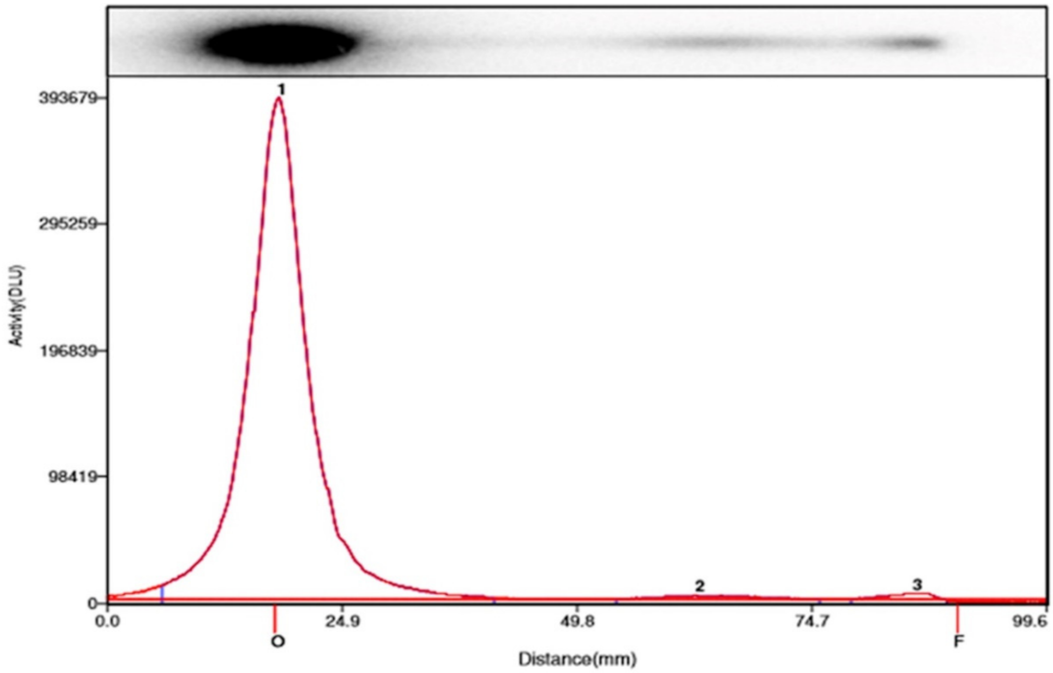

Figure S12. ITLC of raw ^{68}Ga-MacroP with Method 1 (0.1 M sodium citrate buffer), autoradiographic detection.

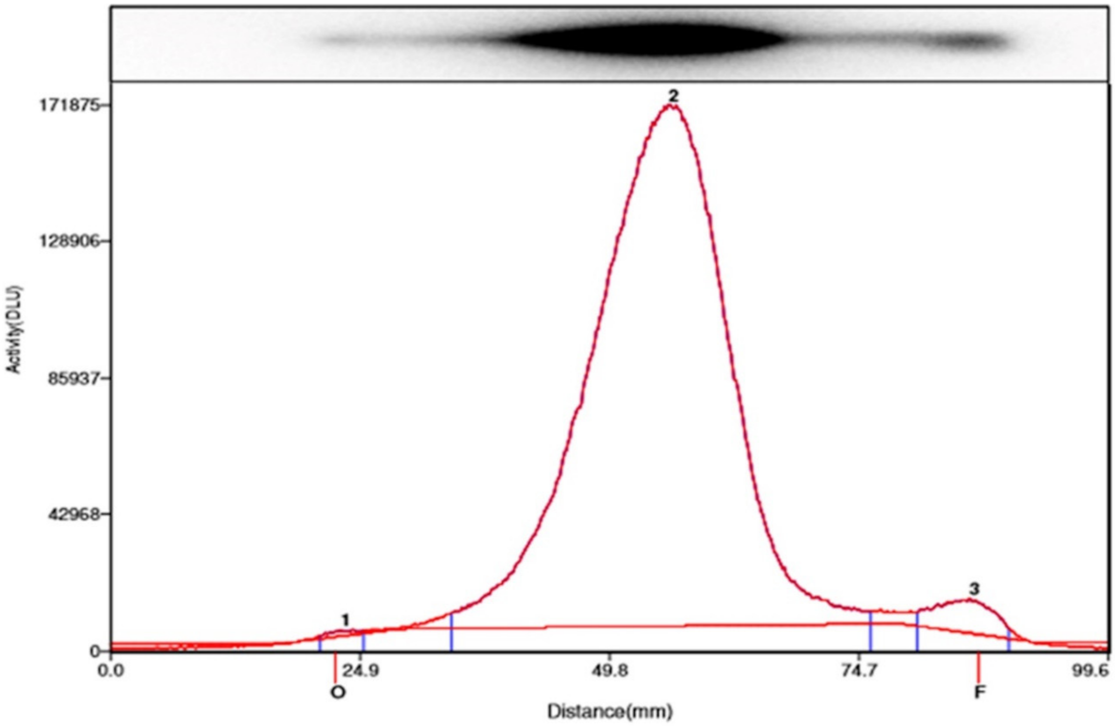

Figure S13. ITLC of raw ^{68}Ga-MacroP with Method 2 (ammonium acetate in water/methanol 1:1), autoradiographic detection.

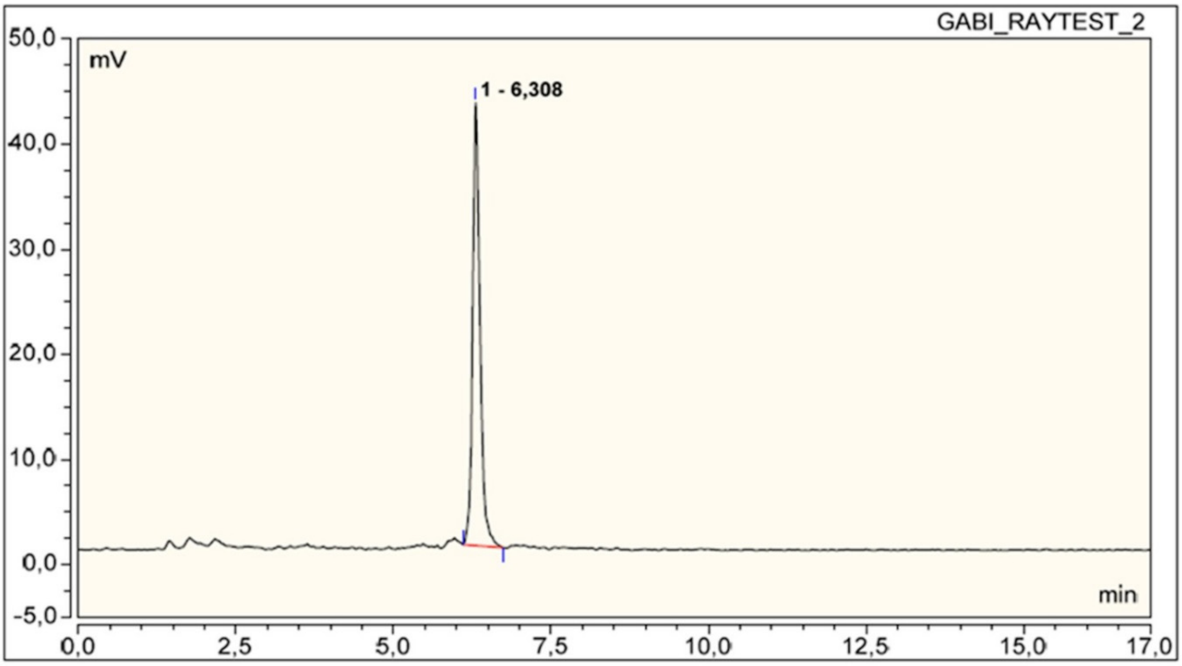

Figure S14. RP-HPLC of pure ^{68}Ga-MacroP, radioactive detector.

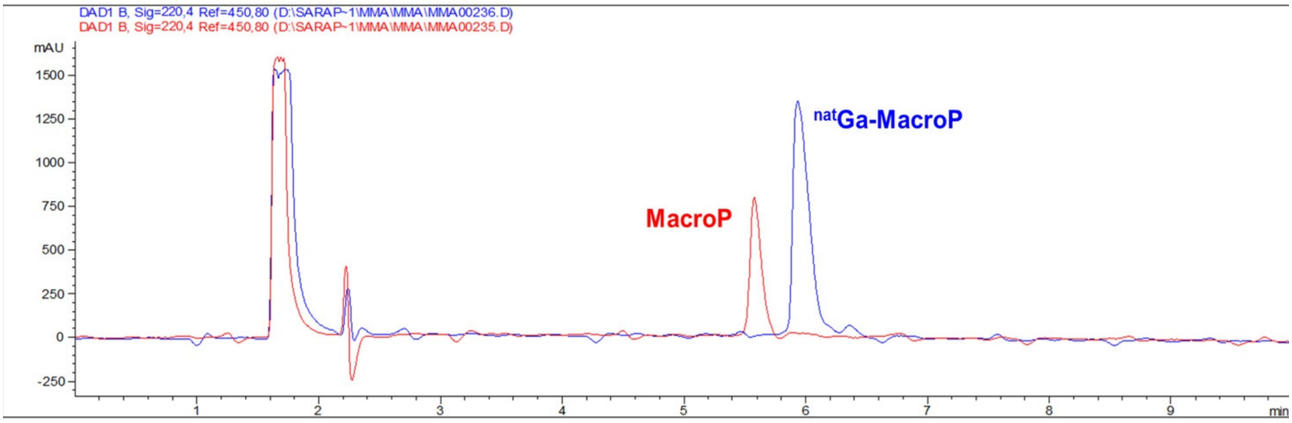

Figure S15. Comparison of RP-HPLC UV (220 nm) chromatograms of MacroP simulated radiolabeling reaction mixture at the beginning (red curve, MacroP, t_R = 5.6 min) and at the end of the reaction time (blue curve, ^{nat}Ga-MacroP, t_R = 6.1 min).

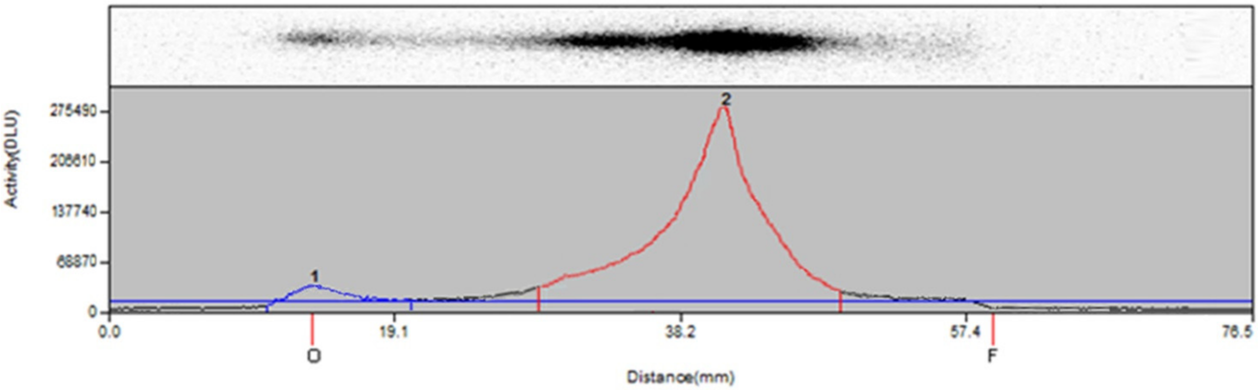

Figure S16. ITLC of ^{68}Ga-BisDOTA. Stationary phase: Whatman MKC18F silica gel plate; mobile phase: 0.9% NaCl:acetonitrile 1:1 (*v*/*v*); autoradiographic detection.

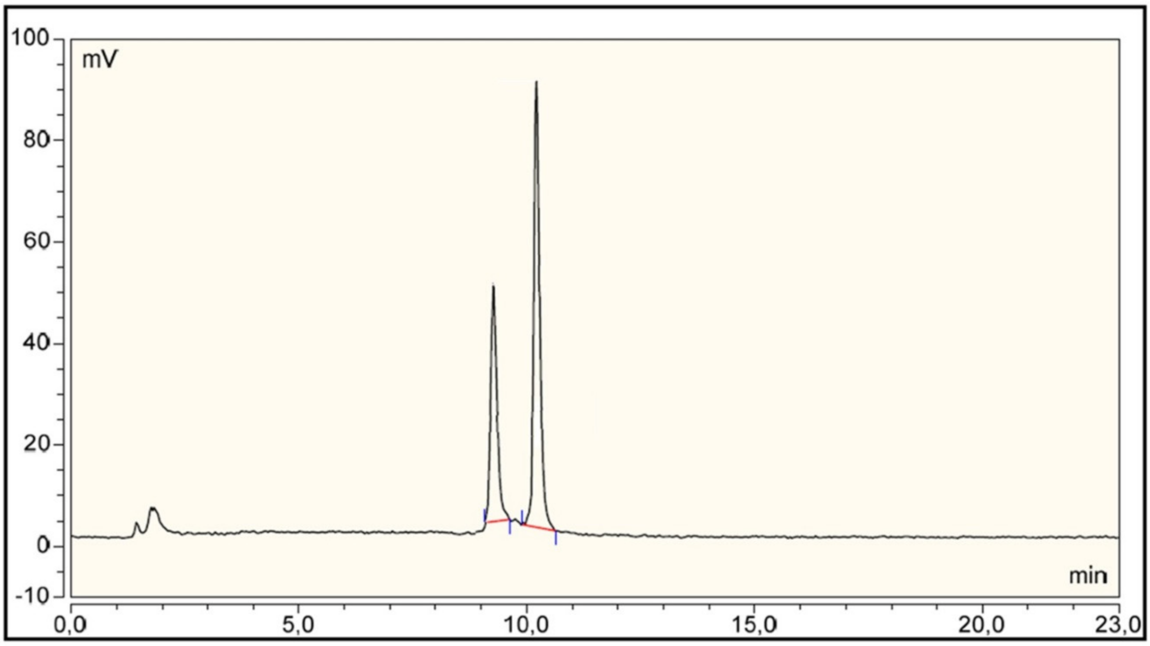

Figure S17. RP-HPLC of ^{68}Ga-BisDOTA, radioactive detector.

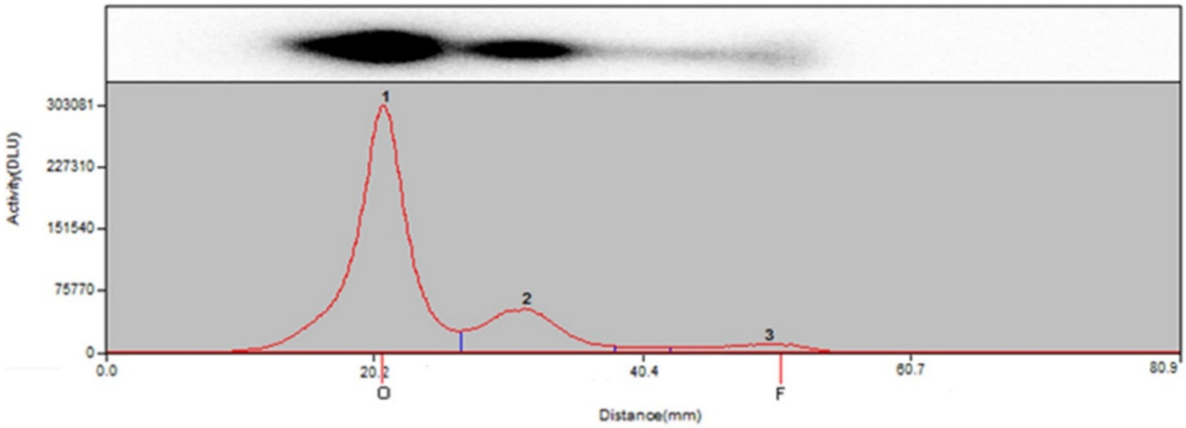

Figure S18. ITLC of NAMP-avidin-^{68}Ga-BisDOTA. Stationary phase: Whatman MKC18F silica gel plate; mobile phase: 0.9% NaCl:acetonitrile 1:1 (*v*/*v*); autoradiographic detection.

Table S4. Stability study of ^{68}Ga-MacroP in saline.

Time (hour)	RCP (%)
0	96.6
2	95.6
3	95.9
4	95.9

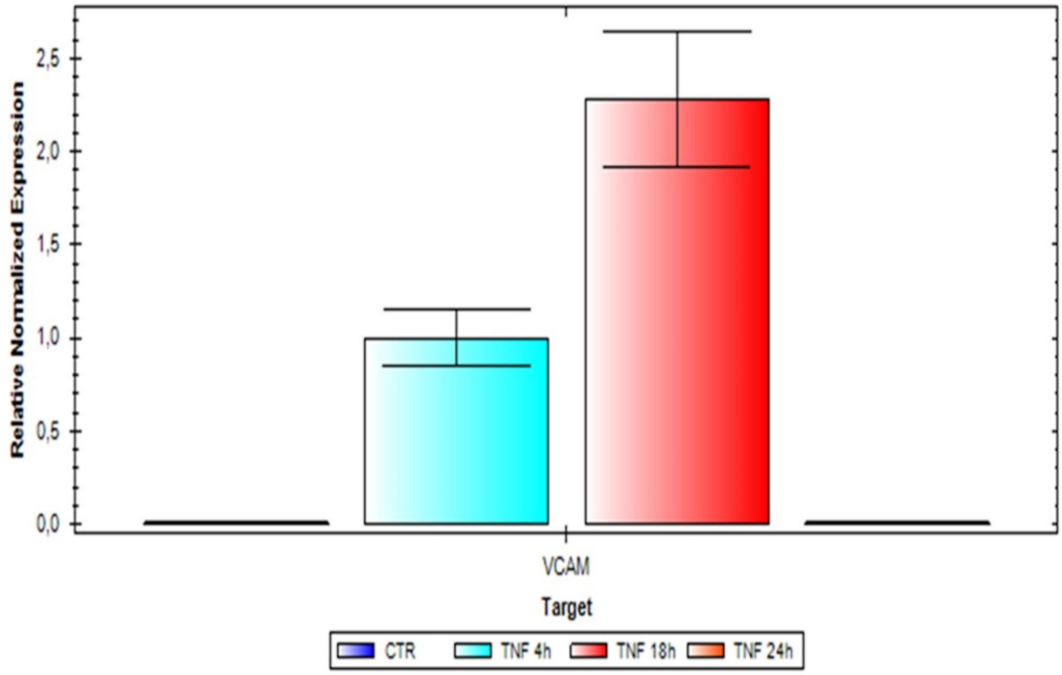

Figure S19. Quantitative PCR analysis showing VCAM-1 mRNA expression on HUVEC before (CTR) and after activation with TNF-α for 4, 18 and 24 h.

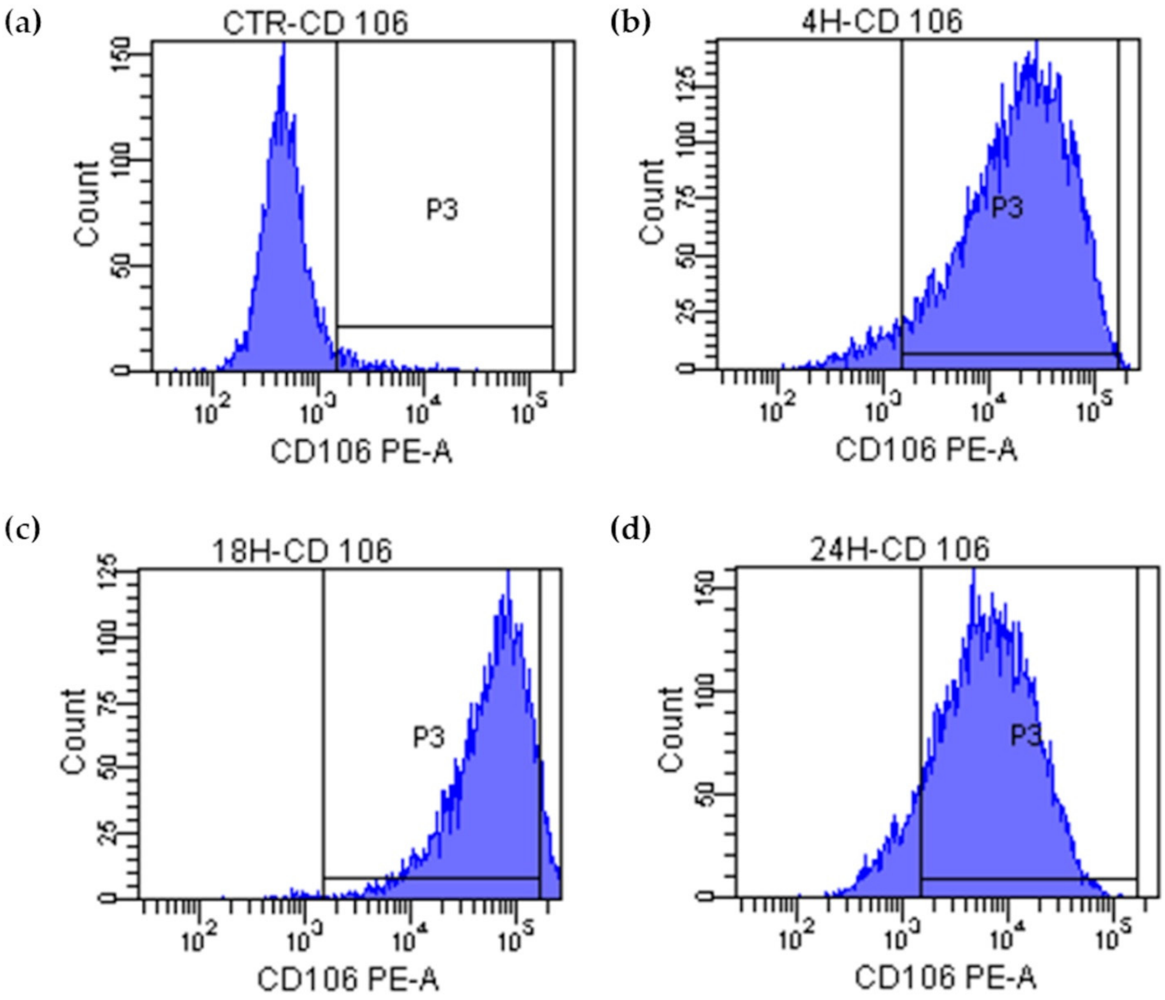

Figure S20. FACS analysis of control cells (**a**) and cells activated with TNF-α for 4 h (**b**), 18 h (**c**) and 24 h (**d**).

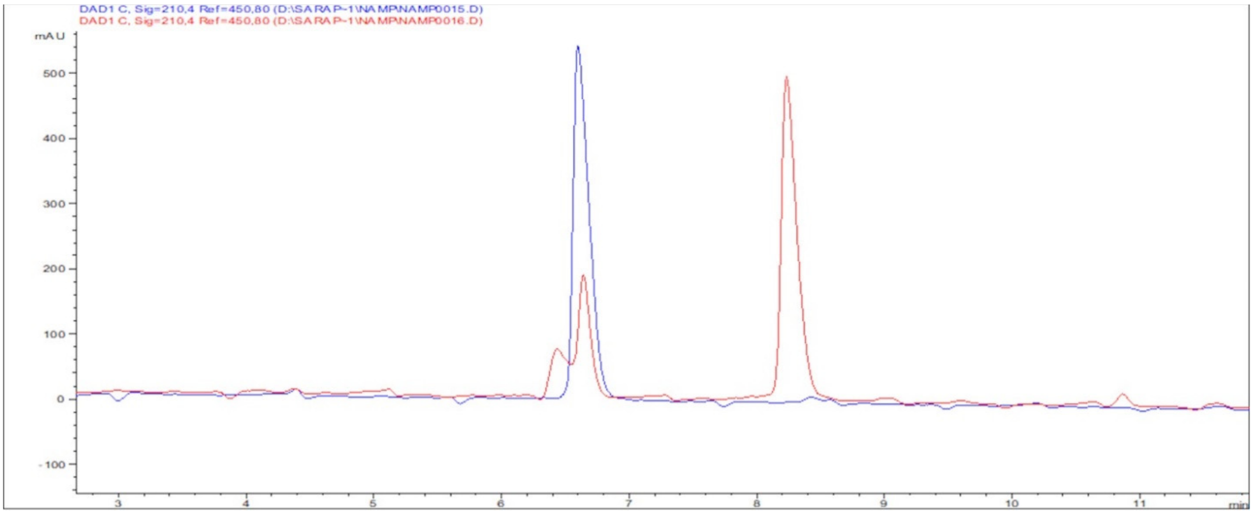

Figure S21. Analytical RP-HPLC comparison between the peptide dissolved in water (blue) and in phosphate buffer (red) after 24 h. The HPLC method was the same as described for NAMP analysis by analytical RP-HPLC.

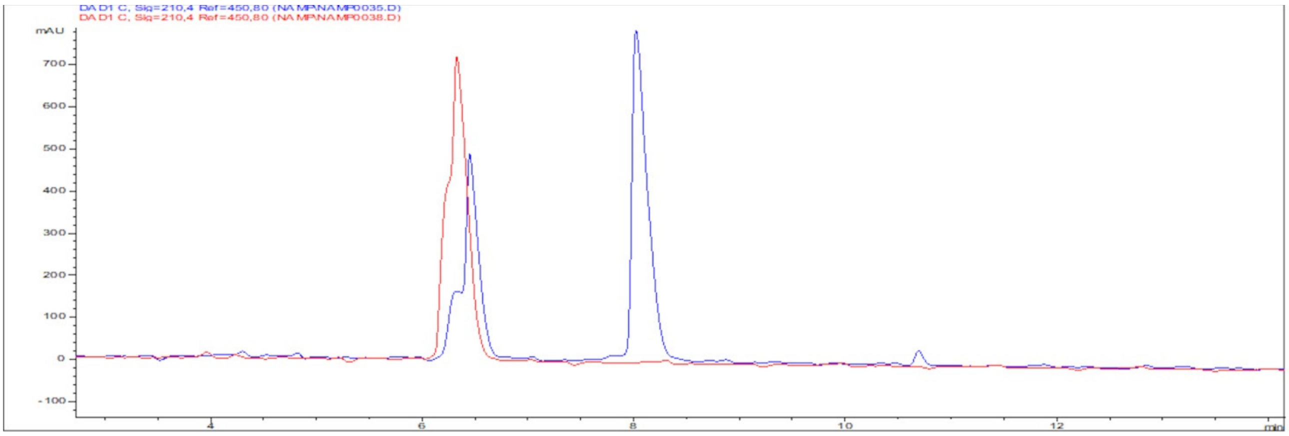

Figure S22. Analytical RP-HPLC comparison between the peptide dissolved in phosphate buffer before (blue) and after (red) TCEP addition.
